# Transcriptome Sequencing and Profiling of Expressed Genes in Phloem and Xylem of Ramie (*Boehmeria nivea* L. Gaud)

**DOI:** 10.1371/journal.pone.0110623

**Published:** 2014-10-29

**Authors:** Jianrong Chen, Fang Liu, Yinghong Tang, Youmei Yuan, Qingquan Guo

**Affiliations:** Department of Biotechnology and Environmental Science, Changsha University, Changsha, Hunan, China; USDA-ARS-SRRC, United States of America

## Abstract

Ramie (*Boehmeria nivea* L. Gaud) is a highly versatile herbaceous plant which is widely cropped in southern China. The success of this herbaceous plant relies on wide use in modern industry. Understanding the profiling of expressed genes in phloem and xylem of ramie is crucial for improving its industrial performance. Herein, we uncover the transcriptome profile in phloem and xylem in present study. Using Illumina paired-end sequencing technology, 57 million high quality reads were generated. De novo assembly yielded 87,144 unigenes with an average length of 635 bp. By sequence similarity searching for public databases, a total of 32,541 (41.77%) unigenes were annotated for their function. Among these genes, 57,873 (66.4%) and 28,678 (32.9%) unigenes were assigned to categories of Gene Ontology and Orthologous Groups database, respectively. By searching against the Kyoto Encyclopedia of Genes and Genomes Pathway database (KEGG), 18,331 (21.0%) unigenes were mapped to 125 pathways. The metabolic pathways were assigned the most unigene (4,793, 26.2%). Furthermore, Pol II and Pol III subunits as well as the genes of Galactose metabolism pathway had higher expression in phloem compared to xylem. In addition, fatty acid metabolism pathway genes showed more abundant in xylem than phloem. These results suggest that high activities of RNA synthesis and Galactose metabolism pathway promises fiber synthesis in phloem. The present study is the initial exploration to uncover the fiber biosynthesis difference between phloem and xylem in ramie through the analysis of deep sequencing data.

## Introduction

Ramie (*Boehmeria nivea*) is one of the most widely cropped herbaceous plants in southern China which provides important natural fiber for industry, including packing materials, fishing nets, filter cloths, industrial fuel and bio-fertilizers [1], [2], [3]. In ramie, as in other plants, phloem and xylem are two different complex tissues which plays crucial role in transporting food and water in plant [4]. In modern industry, these two tissues have different uses. Bast fibers in phloem of ramie are one of the best clothing materials which are the second usage clothing materials in China except cotton fiber [5], [6] while, lignin in xylem is great raw material for industrial ethanol production and suitable material for bio-fertilizers after fermentation [7], [8]. Hence, several studies have been focus on the mechanisms of phloem and xylem development in ramie [6], [9], [10], [11]. These researches focus on single gene or a class of single-function genes expression in phloem and xylem. However, to better understand the differentiation of the net work genes between phloem and xylem, high-throughput technology needs to be employed.

Nowadays, system biology is applied to explore metabolic network of fiber biosynthesis in plants by improving lignocellulose biological quality with the ultimate purposes of biofuel reduction in processing and disposing, cost saving and pollution reduction [12], [13]. Cell behavior and biological phenomena can be comprehended better with the study on metabolic network through analyzing biochemical data, exploring the relations between structure and function of metabolic network as well as the relations between the metabolic flow and gene expression [14]. However, up to date, little information has been given in metabolic network in ramie which could aid to planting industry and machining. In 2013, Liu et al reported a transcriptome analysis using Illumina paired-end sequencing in ramie which is the first time to characterize the ramie transcriptome [15]. Also, they detected the different expression among leaf, root, stem bast, stem xylem and stem shoot. The report offered a new sight for ramie study. Considering the industrial uses of ramie, the most urgent need is to understand the metabolic network of fiber biosynthesis in phloem and xylem. Once understanding the different metabolic network of fiber biosynthesis between these two different tissues, we could adjust the breeding and planting strategy of ramie for higher productive efficiency.

Next-generation sequencing offered a new approach which is more efficient and less cost for obtaining functional genomic information [16]. However, for non-model organisms, high sequencing coverage depth and facilitates the de novo assembly are needed. Furthermore, next-generation sequencing provides a new strategy which promises understanding the gene expression differentiation without knowing sequences. This superiority of next-generation sequencing guarantees transcriptome scanning for network genes rather than micro-arrayed in non-model organisms [17]. Thus, next-generation sequencing is powerful and efficient tools to understand the molecular mechanisms involved in fiber biosynthesis and key targets for genetic manipulation as well as germplasm improvement. In recent years, this technology has been applied in several studies for plant fiber formation and biosynthesis [18], [19], [20]. However, very limited genomics and functional genomics resources which related to the mechanisms of ramie fiber formation has been unveiled.

In the present study, we used the Illumina paired-end sequencing technology to characterize the transcriptome of phloem and xylem tissues in ramie and identified the different expressed genes between phloem and xylem. These data offer useful information for understand the gene controlling of the fiber biosynthesis. To our knowledge, this is the initial exploration to uncover the fiber biosynthesis difference between phloem and xylem in ramie through the analysis of deep sequencing data.

## Materials and Methods

### Materials and RNA extraction

**Figure 1:**
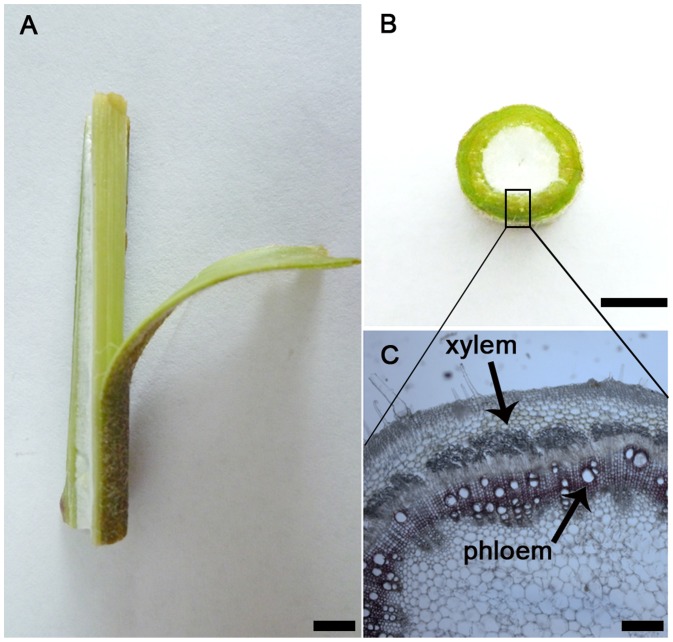
Phloem and xylem in stem of ramie. A. longitudinal view of the ramie stem. Bar = 8 mm. B. cross-sectional view of the ramie stem. Bar = 10 mm. C. tissue section view of the ramie stem. Bar = 0.5 mm.

All the ramie individuals in the present study were grown in the experimental field in Department of Biotechnology and Environmental Science, Changsha University. The samples of ramie were planted in Changsha University, Department of Bioengineering & Environmental Science. We used 5 different adult individuals for this study. The phloem and xylem tissues were distinguished and collected under the stereomicroscope from the middle stem of the plants (Fig. 1). The tissues were immediately frozen in liquid nitrogen and then stored at −80° until total RNA isolation. The Total RNAs were extracted from each tissue of ramie of three different individuals by using Plant DNA Isolation Kit (Invitrogen, CA, USA).

### cDNA library construction and transcriptome sequencing

The cDNA library construction and transcriptome sequencing were performed by BGI-Shenzhen (Shen Zhen, China) using a HiSeq 2000 platform (Illumina, San Diego, CA, USA). Briefly, poly(A) RNA was isolated from 20 µg total RNA with Sera-mag Magnetic Oligo (dT) Beads (Illumina). First, the mRNA was fragmented into small pieces and then the cDNA was synthesis using random hexamer (N6) primers (Illumina) according to the end-repair and phosphorylation protocol. Second, 15 rounds of PCR amplification was used to enrich the end-repair cDNA. Finally, the cDNA library was underwent Illumina sequencing in an Illumina Genome Analyzer HiSeq 2000 according to the manufacturer’s instruction. The sequencing data is available in the NCBI Sequence Read Archive (SRA, http://www.ncbi.nlm.nih.gov/Traces/sra) with accession number of SRP043128.

### Data process and de novo assembly

After transcriptome sequencing, for de novo assembly, all the raw reads were filtered into high-quality clean reads. The clean reads from the cDNA libraries were filtered by low-quality reads and adaptors-only with Q value ≤20. We used Trinity software (http://trinityrnaseq.sourceforge.net/) for de novo assembling with an optimized k-mer length of 25. The paired-end reads were assigned to different contigs for following annotation. We also used TGICL to filter out the redundant unigene and process single set of non-redundant unigene.

### Gene annotation

BLASTx alignment E-value≤10^−5^ was performed to determine the functional annotation of unigenes. The protein databases of BLASTx are non-redundant protein database (nr) form NCBI (http://www.ncbi.nlm.nih.gov), the Cluster of Orthologous Groups database (COG) (http://www.ncbi.nlm.nih.gov/COG), Swiss Prot (http://www.expasy.ch/sprot), Protein Information Resource (PIR) (http://pir.georgetown.edu/), Protein Research Foundation (PRF) (http://www.prf.or.jp/index-e.html) and Protein Data Bank (PDB) (http://www.rcsb.org/) and Kyoto Encyclopedia of Genes and Genomes (KEGG) pathway database (http://www.genome.jp/kegg). The best matched results were regarded as the sequence direction of unigenes. Once the results were different from the different database, we set the priority order of the database as nr, COG, Swiss Prot, PIR, PRF, PDB and KEGG. Once a unigene could not match any sequence in all the databases, we used ESTScan software to predict the coding region and to decide the sequence direction of unigenes.

Subsequently, the unigenes were annotated by Blast2GO program to obtained Gene Ontology (GO) annotation according to the three levels including molecular function, biological process and cellular component ontologies. The GO annotation plot was depict from WEGO website by BGI. The unigenes were assigned into different classify functions by match the genes in COG database. The pathway assignments of the ramie unigenes were predicted by assign into KEGG genes with BLASTx.

### Analysis and screening of differentially expressed genes

After sequence assembly and gene prediction of the cDNA sequences, we used reads count and length information to analyze the differentially expressed genes in phloem and xylem of ramie. To calculate the relative expression levels of phloem and xylem, the reads counts were normalized to the total number of produced reads for each sample and calculated by reads per kb per million reads (RPKM) method. The significance of gene differential expression level was assessed using R, DEGseq package. The significant differentially expressed genes were confirmed from the assay of DEGseq package with *P*≤0.0001 based on false discovery rate.

### Real-time PCR

To validate the expression profiles of the selected genes, real-time PCR was performed. Total RNAs of the tissues were isolated and then cDNA were synthesized using MMLV reverse transcriptase (Invitrogen, Carlsbad, CA, USA). The real-time PCR primers were showed in Table S1. The SYBR Green I Master Mix (TaKaRa, Dalian, China) was used. The reactions were performed as denaturation at 95°C for 5 min; followed by 40 amplification cycles at 95°C for 15 s and 60°C for 30 s. Dissociation protocols were used to measure melting curves. β-actin were used as internal control. The relative expression levels were calculated by 2^−ΔΔCt^.

## Results

### RNA-seq and gene annotation by searching against public databases

**Figure 2:**
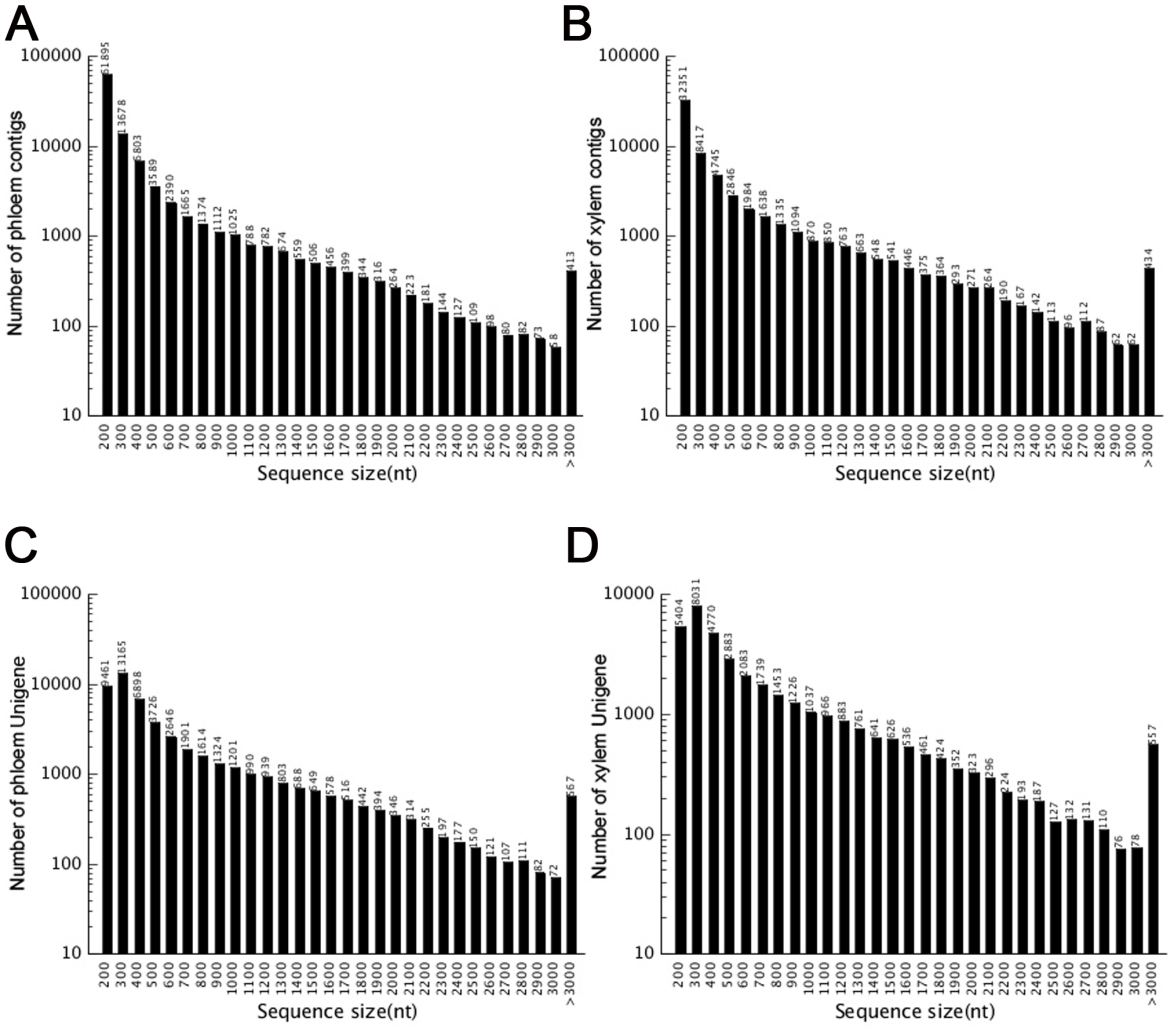
Length distribution of assembled contigs and unigenes. A. length distribution of phloem contigs. B. length distribution of xylem contigs. C. length distribution of phloem unigenes. D. length distribution of xylem unigenes.

**Figure 3:**
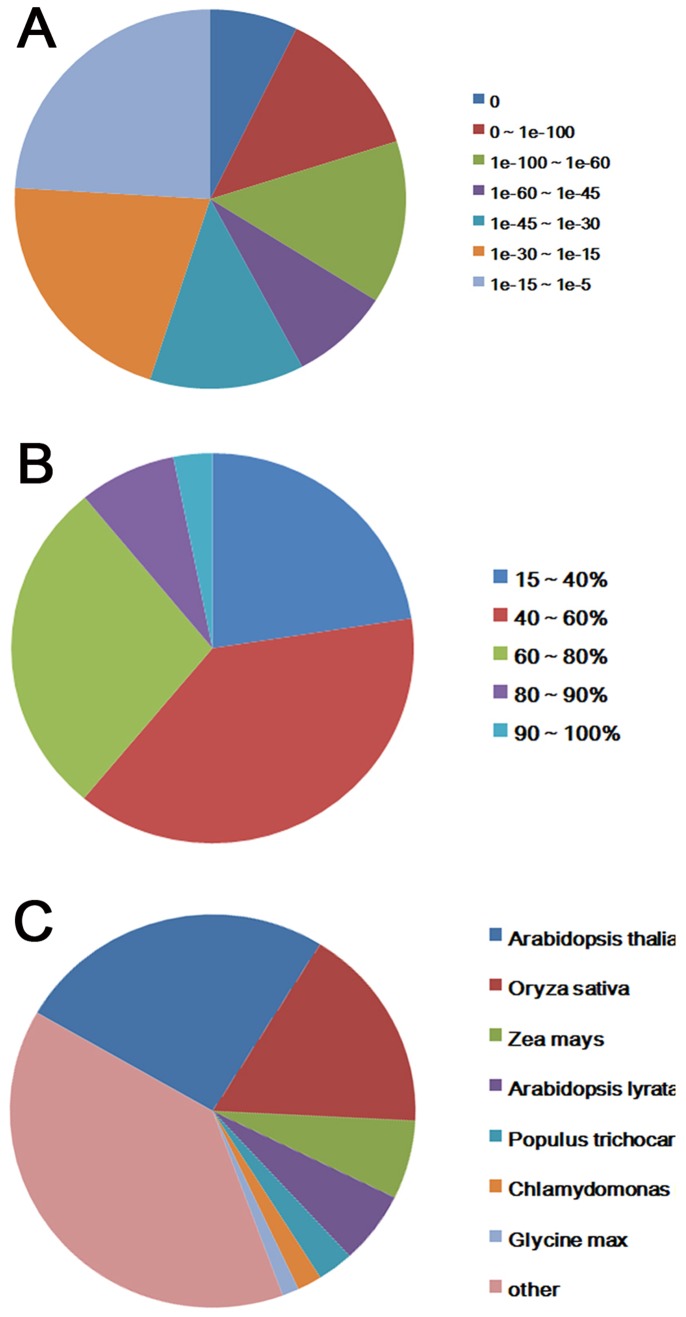
Characteristics of similarity search of unigenes against Nr databases. A. E-value distribution of BLAST hits for each unigene with a cutoff E-value of 1.0E-5. B. Similarity distribution of the top BLAST hits for each unigene. C. Species distribution of the top BLAST hits for each unigenes in Nr dababase.

Sequences of the two cDNA libraries derived from phloem and xylem tissues generated 33.0 and 33.4 million reads, respectively. The total data contained 10 Gb of sequences with average length of 200 bp. After filtered the low-quality reads, approximately 57 million high quality reads were obtained. Based on these high quality reads, 162,330 contigs with an average length of 357 bp ranging from 100 to 14,587 bp were obtained after assembly (Fig. 2). For each tissue, 100,207 and 62,123 contigs were assembled with an average length of 319 and 418 bp in ramie phloem and xylem, respectively. Unigenes were produced by realignment of paired-end reads to contigs, and the contigs in one transcript which assembled with not extended on the end were considered as unigenes. In total, after assembly, 87,144 unigenes were yielded with average length of 635 bp. For phloem and xylem, 50,434 and 36,710 unigenes were obtained, respectively. About 83.88% of the unigenes were produced from more than 10 reads. All the unigenes were predicted via BLAST against public databases. With the E-value threshold of 10^−5^, 32,541 (41.77%) unigenes were matched the sequences in the databases. The E-value distribution of the top hits in the Nr database revealed that the mapped sequences showed significant homology (Fig. 3A), and 38.5% of the sequences with similarities were the 40–60% were found (Fig. 3B). The most target sequences were from *Arabidopsis thaliana* and *Oryza sativa* (Fig. 3C). For phloem and xylem, 15,219 (36.65%) and 17,322 (47.61%) unigenes were annotated from the databases, respectively (Table S2).

### GO and COG analysis of the unigenes

**Figure 4:**
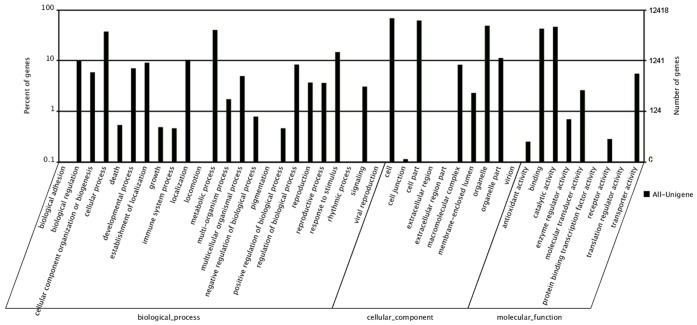
Gene Ontology classifications of assembled unigenes.

Gene Ontology (GO) as a standardized gene functional classification system has been used for illustrating the properties of genes which is widely used in transcriptome and micro-array analysis. Three different ontologies were set up, including molecular function, cellular component and biological process. In present study, 57,873 (66.4%) unigenes were assigned to GO classes with 5,783 functional terms. Of these unigenes, 20,141 (23.1%), 24,819 (28.5%) and 12,913 (14.8%) were assigned into Molecular function, Cellular component and Biological process ontologies, respectively (Fig. 4).

**Figure 5:**
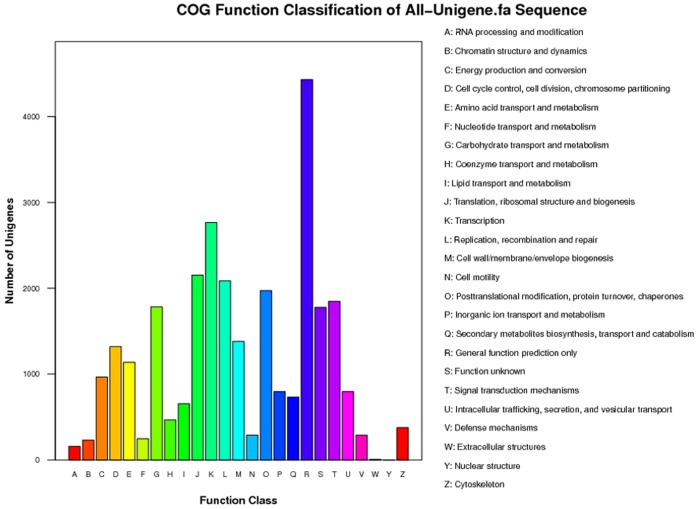
Histogram presentation of clusters of orthologous groups (COG) classification.

Also, we used COG for classify of orthologous gene products. Totally, 28,678 genes (32.9%) were assigned to the 25 COG classifications (Fig. 5). Among all the COG groups, the cluster of General function prediction represented the largest groups which had 4,431 (15.5%) unigenes. Translation, ribosomal structure and biogenesis cluster 2,153 (7.5%), Transcription cluster 2,766, (9.6%) and Replication, recombination and repair cluster 2,087 (7.3%) were following the General function prediction. The Extracellular structure cluster and nuclear structure cluster only had few unigenes in the groups (Fig. 5).

### Metabolic pathway analysis by KEGG

The database of KEGG represents the knowledge on the molecular interaction and reaction network. Pathway-based analysis would aid us to understand the interactions among the high-throughput genes. After BLASTx with E-value threshold of 10^−5^, 18,331 (21.0%) unigenes were assigned to 125 KEGG pathways. The pathway of Metabolic pathway was assigned the most unigene (4,793, 26.2%), followed by Biosynthesis of secondary metabolites (2,071, 11.3%), RNA transport (1,256, 6.9%), Plant-pathogen interaction (1,201, 6.6%), Endocytosis (1,104, 6.0%), Glycerophospholipid metabolism (1,047, 5.7%), Plant hormone signal transduction (973, 5.3%), Ether lipid metabolism (954, 5.2%), mRNA surveillance pathway (864, 4.7%), Spliceosome (805, 4.4%), whereas only no more than 10 unigenes were assigned to Synthesis and degradation of ketone bodies, C5-Branched dibasic acid metabolism, Anthocyanin biosynthesis, Fatty acid elongation, Biotin metabolism, Lipoic acid metabolism, Lipoic acid metabolism and Caffeine metabolism (Table S3).

### Differential gene expression between phloem and xylem

**Figure 6:**
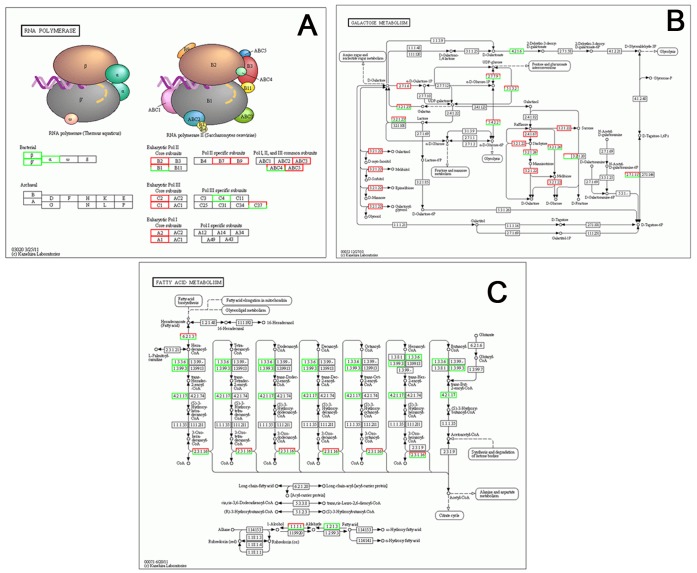
Pathways of ramie differentially regulated in phloem and xylem. A. Graphic representation of the global changes in RNA polymerase pathway. B. Graphic representation of the global changes in galactose metabolism pathway. C. Graphic representation of the global changes fatty acid metabolism pathway. Red and green boxes indicate the up-regulated and down-regulated genes when phloem compared to xylem tissues.

To determine the differential gene expression between phloem and xylem, we first prepare the cDNA libraries with standard Illumina random-prime PCR technique to enrich mRNA of the samples and then used DEGseq package of R to examine differential gene expression. The differential expression was defined as the expression fold of gene specific mRNA levels with *P* value ≤0.0001. In total, 10,870 unigenes were found that significantly higher expressed in phloem tissue while only 4,440 unigenes were higher expressed in xylem tissue compared with phloem tissue. Interestingly, most of the genes classified in RNA polymerase pathway show an increased expression in phloem, including Pol II Core subunits B2, Pol II specific subunits B7, Pol II specific subunits B9, Pol common subunits ABC3, Pol common subunits ABC5, Pol III Core subunits C1, Pol III Core subunits C2 (Fig. 6A) (Table S4, S5). These genes encode well-described RNA polymerase pathway genes. Meanwhile, Galactose metabolism pathway increased in xylem tissue compared with phloem tissue (Fig. 6B) which showed stimulation of polysaccharides synthesis. On the other hand, most of the genes classified in fatty acid metabolism pathway show a decreased expression in phloem, including ACOX1, ACOX3, ACADM, paaF, and ECHS1 (Fig. 6C) (Table S4, S5). It is surprising that higher expression of fatty acid metabolism pathway which could produce more energy.

**Figure 7:**
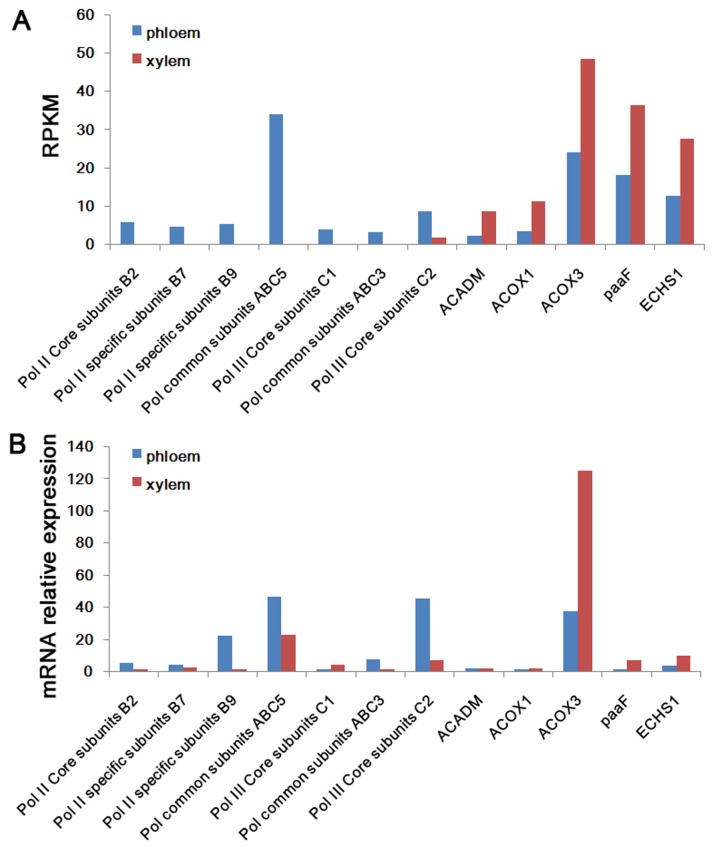
Confirmation of differential expression between phloem and xylem by real-time PCR. A. Different expression of Pol II Core subunits B2, Pol II specific subunits B7, Pol II specific subunits B9, Pol common subunits ABC3, Pol common subunits ABC5, Pol III Core subunits C1, Pol III Core subunits C2, ACOX1, ACOX3, ACADM, paaF, and ECHS1 by transcriptome sequencing using RPKM method. B. Different expression of Pol II Core subunits B2, Pol II specific subunits B7, Pol II specific subunits B9, Pol common subunits ABC3, Pol common subunits ABC5, Pol III Core subunits C1, Pol III Core subunits C2, ACOX1, ACOX3, ACADM, paaF, and ECHS1 by real-time PCR.

To confirm the expression differentiation between phloem and xylem, real-time PCR was employed. The expression levels of Pol II Core subunits B2, Pol II specific subunits B7, Pol II specific subunits B9, Pol common subunits ABC3, Pol common subunits ABC5, Pol III Core subunits C1, Pol III Core subunits C2, ACOX1, ACOX3, ACADM, paaF, and ECHS1 were further examined (n = 5). The results showed that the results of RPKM are similar with the results of real-time PCR. The gene expression of Pol II Core subunits B2, Pol II specific subunits B7, Pol II specific subunits B9, Pol common subunits ABC3, Pol common subunits ABC5, Pol III Core subunits C1, Pol III Core subunits C2 were up-regulated in phloem while ACOX1, ACOX3, ACADM, paaF, and ECHS1 showed contrary results (Fig. 7).

## Discussion

The strict controlled sequential changes of gene expression are required for tissue differentiation during plant development. In previous studies, enormous surveys of transcriptional regulation of tissue differentiation were conducted using microarray and EST sequencing [21], [22], [23]. In most recent studies, transcriptomes of different tissues by RNA-seq have been conducted. For the most part, these data is limited in the connection between productive performance and RNA sampled from a variety of tissues. Up to date, few studies have focused on the gene expression profiles of ramie. This is because ramie is a non-model organism and small planting areas all over the world. To our knowledge, the present research is the first study to explore the global gene expression profiles of in phloem and xylem of ramie. In ramie, phloem and xylem are used for clothing materials and renewable resources, respectively [24]. However, there are currently no comprehensive sequences or comparative transcriptomic analyses of phloem and xylem in ramie by RNA-seq. The present study provides large amount of sequences in these tissues. Of all the clean reads, 58.23% were not matched to any specific genes. The unmapped reads may for the following reasons: 1) the reads were not recognized by the genomes from databases; 2) sequencing errors lead to fail assemble; 3) alternatively spliced exons of the short reads resulted in failure matching of the reference genome; 4) several reads were from the contaminated genomes, such as microbes; 5) sequence differences between trancriptomes of ramie and reference genome.

Recently, a study characterized transcriptomes of 5 mixed tissues in ramie, including leaf, root, stem bast, stem xylem and stem shoot [15]. They characterized 43,990 genes from the ramie transcriptome and provided information of ramie growth and fiber development, which was aid to understanding of ramie growth and fiber development. This work extended our knowledge in understanding of expression profiles, regulation and networks of important traits of the ramie. However, the details of the most valuable productive tissues, such as phloem and xylem, were not distinguished. In our study, the 15,219 (36.65%) and 17,322 (47.61%) genes expressed in the phloem and xylem reported herein represent a large and previously transcriptomic resource for ramie. Also, the transcriptomic of two important tissues were distinguished. For all the genes, approximately 41.77% of the unigenes discovered in this study was ascertained. These genes were annotated from the public database which exhibited their function. Furthermore, these unigenes were assigned into different COG, GO and KEGG groups or pathways. These data will provide clues which would aid to explore the major genes for important agronomic traits and molecular mechanism of fibrogenesis in ramie.

The transcript profiling of phloem and xylem has been reported in several plants. By subtractive library method, Foucart and colleagues identified 263 unique sequences by comparing phloem to xylem [25]. However, due to the limited quantity of the data by subtractive library sequencing, the overall scanning of transcript profiling needs to be provided. Microarray technology may be one of the most useful resolutions. Zhao and colleagues studied xylem and phloem transcriptomes in Arabidopsis Root-Hypocotyl using Affymetrix GeneChip which promised large amount of data at one time [26]. They found that class III, HD, ZIP and KANADI transcription factors are high expressed during secondary growth which demonstrated that the up-regulation of these factors are key evidence in regulating of xylem or phloem cell differentiation and activity. Compared to traditional ESTs scanning and microarray technology, RNA-seq provides a more effective and economic way to investigate the profile of tissues transcriptomes. First, RNA-seq could obtain enormous data in one experiment which is similar with microarray; second, RNA-seq also aid to obtain new transcripts while microarray could not. In present study, we successfully obtained large sequences in phloem and xylem cDNA pools, and the differential expression genes are also defined. Thus, the results proved that RNA-seq is an effective and useful protocol to investigate the expression profiles of phloem and xylem.

The identification and characterization of the phloem and xylem enhanced genes in ramie is of vital importance to our understanding how these molecular differences regulate phloem and xylem differentiation and development. Some of the phloem enhanced genes were found. These genes included the following: (1) Pol II and Pol III subunits, which has been characterized to be required for RNA polymerization [27], [28]; (2) Galactose metabolism pathway genes which participates polysaccharides synthesis [29]. On the other hand, some of the xylem-enhanced genes have been proven to be higher expression compared to phloem. These representative genes include the following: ACOX1, ACOX3, ACADM, paaF, and ECHS1 which participates in fatty acid metabolism pathway [30], [31]. From this point of sight, the transcriptomic analysis of ramie is indeed an economic and efficient approach to identify tissue-enhanced genes. Further functional characterization of these genes should be focus on molecular mechanisms of these genes during development in ramie. Herein, we speculate that the enhanced expressed genes in phloem or xylem triggers the metabolic function in each tissue. The differential expression genes are functionally expressed in phloem and xylem tissues. For phloem enhanced genes, RNA polymerization indicates the high transcriptional activity. Considering phloem is the main location for primary metabolism of ramie, it is no surprise to understand high expression of these genes compare to xylem. Similarly, up-regulation Galactose metabolism pathway promises fiber synthesis in phloem. Intriguing, fatty acid metabolism pathway was up-regulated in xylem. This result suggested that more energy production were in xylem. In ramie, the connection of galactose and/or fatty-acid metabolism and the agronomic properties are still limited. Thus, the details and its influence are needed to be elucidated in further researches.

In conclusion, the present study provides a portfolio of candidate genes for future studies on fibre formation. However, the exact roles of these specific expressed genes in differentiation of ramie tissues are still unveiled. One of the possible roles of these specific expressed genes may be to antagonize the genes responsible for the other key pathway for specific tissues differentiation involved in development. In this way, further researches needs to be characterized the function of these genes and illustrated the paradigm during different developmental stages.

## Supporting Information

Table S1 **Primers used in the present study.**
(DOC)

Table S2 **Transcripts annotation of phloem and xylem in ramie.**
(XLSX)

Table S3 **Pathway assignment based on KEGG of Transcripts annotation of phloem and xylem in ramie.**
(DOCX)

Table S4 **Different expression analysis between phloem and xylem in ramie.**
(XLSX)

Table S5 **Pathway assignment of DEGs genes based on KEGG.**
(DOCX)

## References

[1] Liu LJ, Chen HQ, Dai XB, Wang H, Peng DX (2012) Effect of Planting Density and Fertilizer Application on Fiber Yield of Ramie (*Boehmeria nivea*). J Integr Agric 11: 1199–1206.

[2] Liu X, Chen J, Sun P, Liu ZW, Liu ZT (2010) Grafting modification of ramie fibers with poly(2,2,2-trifluoroethyl methacrylate) via reversible addition-fragmentation chain transfer (RAFT) polymerization in supercritical carbon dioxide. React Funct Polym 70: 972–979.

[3] Mukhopadhyay A, Dutta N, Chattopadhyay D, Chakrabarti K (2013) Degumming of ramie fiber and the production of reducing sugars from waste peels using nanoparticle supplemented pectate lyase. Bioresour Technol 137: 202–208. 23587821 10.1016/j.biortech.2013.03.139

[4] Carlsbecker A, Helariutta Y (2005) Phloem and xylem specification: pieces of the puzzle emerge. Curr Opin Plant Biol 8: 512–517. 16039153 10.1016/j.pbi.2005.07.001

[5] Angelini LG, Lazzeri A, Levita G, Fontanelli D, Bozzi C (2000) Ramie (*Boehmeria nivea* (L.) Gaud.) and Spanish Broom (*Spartium junceum* L.) fibres for composite materials: agronomical aspects, morphology and mechanical properties. Ind Crops Prod 11: 145–161.

[6] Liu L, Lao C, Zhang N, Chen H, Deng G, et al (2013) The effect of new continuous harvest technology of ramie (*Boehmeria nivea* L. Gaud.) on fiber yield and quality. Ind Crops Prod 44: 677–683.

[7] Angelini LG, Tavarini S (2013) Ramie [*Boehmeria nivea* (L.) Gaud.] as a potential new fibre crop for the Mediterranean region: Growth, crop yield and fibre quality in a long-term field experiment in Central Italy. Ind Crops Prod 51: 138–144.

[8] Liu F, Liang X, Zhang N, Huang Y, Zhang S (2001) Effect of growth regulators on yield and fiber quality in ramie (*Boehmeria nivea* (L.) Gaud.), China grass. Field Crop Res 69: 41–46.

[9] Roy S, Lutfar LB (2012) 4 - Bast fibres: ramie. In: Kozłowski RM, editor. Handbook of Natural Fibres: Woodhead Publishing. 47–55.

[10] Liu F, Huang Y, Guo Q, Zhang X, Li L, et al (2008) Cloning and Expression of UDP-glucose Dehydrogenase (UDPGDH) cDNA in Ramie (*Boehmeria nivea* (Linn.) Gaud.). Scientia Agricultura Sinica 11: 015.

[11] Liu JX, Yu CM, Tang SW, Zhu AG, Wang YZ, et al (2009) Cloning and Expression of Key Enzyme Gene GalAT in Ramie Pectin Biosynthesis. Agric Sci China 8: 664–670.

[12] Jeffries TW, Grigoriev IV, Grimwood J, Laplaza JM, Aerts A, et al (2007) Genome sequence of the lignocellulose-bioconverting and xylose-fermenting yeast Pichia stipitis. Nat Biotechnol 25: 319–326. 17334359 10.1038/nbt1290

[13] Hill J, Nelson E, Tilman D, Polasky S, Tiffany D (2006) Environmental, economic, and energetic costs and benefits of biodiesel and ethanol biofuels. Proc Natl Acad Sci USA 103: 11206–11210. 16837571 10.1073/pnas.0604600103 PMC1544066

[14] Hartwell LH, Hopfield JJ, Leibler S, Murray AW (1999) From molecular to modular cell biology. Nature 402: C47–C52. 10591225 10.1038/35011540

[15] Liu T, Zhu S, Tang Q, Chen P, Yu Y, et al (2013) De novo assembly and characterization of transcriptome using Illumina paired-end sequencing and identification of CesA gene in ramie (*Boehmeria nivea* L. Gaud). BMC Genomics 14: 125. 23442184 10.1186/1471-2164-14-125 PMC3610122

[16] Schuster SC (2007) Next-generation sequencing transforms today’s biology. Nature 200. 10.1038/nmeth1156 18165802

[17] Wall PK, Leebens-Mack J, Chanderbali AS, Barakat A, Wolcott E, et al (2009) Comparison of next generation sequencing technologies for transcriptome characterization. BMC Genomics 10: 347. 19646272 10.1186/1471-2164-10-347 PMC2907694

[18] Eriksson ME, Israelsson M, Olsson O, Moritz T (2000) Increased gibberellin biosynthesis in transgenic trees promotes growth, biomass production and xylem fiber length. Nat Biotechnol 18: 784–788. 10888850 10.1038/77355

[19] Ehlting J, Mattheus N, Aeschliman DS, Li E, Hamberger B, et al (2005) Global transcript profiling of primary stems from Arabidopsis thaliana identifies candidate genes for missing links in lignin biosynthesis and transcriptional regulators of fiber differentiation. Plant J 42: 618–640. 15918878 10.1111/j.1365-313X.2005.02403.x

[20] Zhong R, Burk DH, Ye ZH (2001) Fibers. A model for studying cell differentiation, cell elongation, and cell wall biosynthesis. Plant Physiol 126: 477–479. 11402177 10.1104/pp.126.2.477 PMC1540113

[21] Wang Z, Gerstein M, Snyder M (2009) RNA-Seq: a revolutionary tool for transcriptomics. Nat Rev Genet 10: 57–63. 19015660 10.1038/nrg2484 PMC2949280

[22] Liu T, Zhu S, Tang Q, Yu Y, Tang S (2013) Identification of drought stress-responsive transcription factors in ramie (*Boehmeria nivea* L. Gaud). BMC Plant Biol 13: 130. 24020723 10.1186/1471-2229-13-130 PMC3846573

[23] Wang X, Wang B, Liu L, Cui X, Yang J, et al (2010) Isolation of high quality RNA and construction of a suppression subtractive hybridization library from ramie (*Boehmeria nivea* L. Gaud.). Mol Biol Rep 37: 2099–2103. 19680788 10.1007/s11033-009-9671-7

[24] Dahlke B, Larbig H, Scherzer H, Poltrock R (1998) Natural fiber reinforced foams based on renewable resources for automotive interior applications. J Cellular plast 34: 361–379.

[25] Foucart C, Paux E, Ladouce N, San Clemente H, Grima Pettenati J, et al (2006) Transcript profiling of a xylem vs phloem cDNA subtractive library identifies new genes expressed during xylogenesis in Eucalyptus. New Phytol 170: 739–752. 16684235 10.1111/j.1469-8137.2006.01705.x

[26] Zhao C, Craig JC, Petzold HE, Dickerman AW, Beers EP (2005) The xylem and phloem transcriptomes from secondary tissues of the Arabidopsis root-hypocotyl. Plant Physiol 138: 803–818. 15923329 10.1104/pp.105.060202 PMC1150398

[27] Borchert GM, Lanier W, Davidson BL (2006) RNA polymerase III transcribes human microRNAs. Nat Struct Mol Biol 13: 1097–1101. 17099701 10.1038/nsmb1167

[28] Kimura M, Ishiguro A, Ishihama A (1997) RNA polymerase II subunits 2, 3, and 11 form a core subassembly with DNA binding activity. J Biol Chem 272: 25851–25855. 9325316 10.1074/jbc.272.41.25851

[29] Kalckar HM (1965) Galactose Metabolism and Cell “Sociology”. Science 150: 305–313. 5319435 10.1126/science.150.3694.305

[30] Ohlrogge JB (1994) Design of new plant products: engineering of fatty acid metabolism. Plant Physiol 104: 821. 12232128 10.1104/pp.104.3.821 PMC160678

[31] Weber H (2002) Fatty acid-derived signals in plants. Trends Plant Sci 7: 217–224. 11992827 10.1016/s1360-1385(02)02250-1

