# Correction: Transcriptome Sequencing and Profiling of Expressed Genes in Phloem and Xylem of Ramie (*Boehmeria nivea* L. Gaud)

**DOI:** 10.1371/journal.pone.0117896

**Published:** 2015-02-13

**Authors:** The PLOS ONE Staff

Figure 1 is labelled incorrectly. The authors have provided a corrected version here.

**Figure 1:**
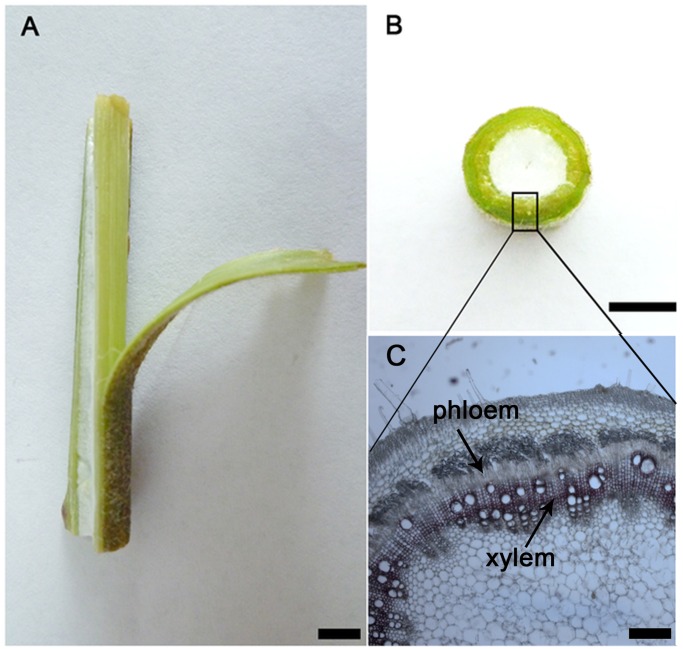
Phloem and xylem in stem of ramie. A. longitudinal view of the ramie stem. Bar = 8 mm. B. cross-sectional view of the ramie stem. Bar = 10 mm. C. tissue section view of the ramie stem. Bar = 0.5 mm. doi:10.1371/journal.pone.0110623.g001

## References

[1] Chen J, Liu F, Tang Y, Yuan Y, Guo Q (2014) Transcriptome Sequencing and Profiling of Expressed Genes in Phloem and Xylem of Ramie (*Boehmeria nivea* L. Gaud). PLoS ONE 9(10): e110623 doi: 10.1371/journal.pone.0110623 25354139 10.1371/journal.pone.0110623 PMC4213010

