# Supplementary material for: Transcriptome Sequencing and Profiling of Expressed Genes in Phloem and Xylem of Ramie (Boehmeria nivea L. Gaud)
Source: PLoS One. 2014 Oct 29;9(10):e110623. doi: 10.1371/journal.pone.0110623 (PMC4213010; doi:10.1371/journal.pone.0110623)
Supplement: Table S1 — Primers used in the present study. (DOC) [file pone.0110623.s001.doc]

| Gene names | Sense (5’ to 3’) | Antisense (5’ to 3’) |
| --- | --- | --- |
| Pol II specific subunits B2 | GGGTCCCCTTCCCTCATCA | AACAAGAGTTCAGGTCGTCAGCA |
| Pol II specific subunits B7 | AGTATGAGTTCCTCAAGGTCTCCG | ATGCCCACGCCAGTTCG |
| Pol II specific subunits B9 | TCGTACCCACAAGAAAGAACAGAA | GCAGCAAGAGCAGACCCAGT |
| Pol common subunits ABC5 | TACCATCAACCCTTCCACCG | GGGCAATCTTCCAGGCAAAC |
| Pol III Core subunits C1 | CTTACGGTGGTGCGGGTGT | CGAGCGGCGTATCCTGACTA |
| Pol common subunits ABC3 | AGTATGAGTTCCTCAAGGTCTCCG | ATGCCCACGCCAGTTCG |
| Pol III Core subunits C2 | GTTCTCCTCTATGCCTGCCGTAA | TCCTCACCCGTAGCACCTCC |
| ACADM | AGTCATCGGGAAATTAGTACAGCA | TTCCCGATGACTTGCCAAAC |
| ACOX1 | CGATTGTTACCAACGGGCTCT | CCAACATTTGCTACCACTCCTGAA |
| ACOX3 | CGGCAAGGTAATTGGGAACA | CGGCAAGGTAATTGGGAACA |
| paaF | CGGCAAGGTAATTGGGAACA | AGTGACAGGTGCTCCATTCCA |
| ECHS1 | GCAAAGAACTGTGACAACTCGTG | CAAAAGCCTCGTCATAATCAAGC |

Table S1 Primers used in the present study.
