# Supplementary material for: Transcriptome Sequencing and Profiling of Expressed Genes in Phloem and Xylem of Ramie (Boehmeria nivea L. Gaud)
Source: PLoS One. 2014 Oct 29;9(10):e110623. doi: 10.1371/journal.pone.0110623 (PMC4213010; doi:10.1371/journal.pone.0110623)
Supplement: Table S3 — Pathway assignment based on KEGG of Transcripts annotation of phloem and xylem in ramie. (DOCX) [file pone.0110623.s003.docx]

Table S2. Pathway assignment based on KEGG.

| Rank | Pathway | Counts | Pathway ID |
| --- | --- | --- | --- |
| 1 | [Metabolic pathways](file:///C:\Documents%20and%20Settings\Administrator\%25E6%25A1%258C%25E9%259D%25A2\%25E9%2599%2588%25E5%25BB%25BA%25E8%258D%25A3\%25E5%259F%25BA%25E5%259B%25A0%25E7%25BB%2584%25E6%2595%25B0%25E6%258D%25AE\%25E5%259F%25BA%25E5%259B%25A0%25E7%25BB%2584%25E6%2595%25B0%25E6%258D%25AE\HSZP10089\annotation\KEGG\All-Unigene.fa.htm#gene1) | 4793 | ko01100 |
| 2 | [Biosynthesis of secondary metabolites](file:///C:\Documents%20and%20Settings\Administrator\%25E6%25A1%258C%25E9%259D%25A2\%25E9%2599%2588%25E5%25BB%25BA%25E8%258D%25A3\%25E5%259F%25BA%25E5%259B%25A0%25E7%25BB%2584%25E6%2595%25B0%25E6%258D%25AE\%25E5%259F%25BA%25E5%259B%25A0%25E7%25BB%2584%25E6%2595%25B0%25E6%258D%25AE\HSZP10089\annotation\KEGG\All-Unigene.fa.htm#gene2) | 2071 | ko01110 |
| 3 | [RNA transport](file:///C:\Documents%20and%20Settings\Administrator\%25E6%25A1%258C%25E9%259D%25A2\%25E9%2599%2588%25E5%25BB%25BA%25E8%258D%25A3\%25E5%259F%25BA%25E5%259B%25A0%25E7%25BB%2584%25E6%2595%25B0%25E6%258D%25AE\%25E5%259F%25BA%25E5%259B%25A0%25E7%25BB%2584%25E6%2595%25B0%25E6%258D%25AE\HSZP10089\annotation\KEGG\All-Unigene.fa.htm#gene3) | 1256 | ko03013 |
| 4 | [Plant-pathogen interaction](file:///C:\Documents%20and%20Settings\Administrator\%25E6%25A1%258C%25E9%259D%25A2\%25E9%2599%2588%25E5%25BB%25BA%25E8%258D%25A3\%25E5%259F%25BA%25E5%259B%25A0%25E7%25BB%2584%25E6%2595%25B0%25E6%258D%25AE\%25E5%259F%25BA%25E5%259B%25A0%25E7%25BB%2584%25E6%2595%25B0%25E6%258D%25AE\HSZP10089\annotation\KEGG\All-Unigene.fa.htm#gene4) | 1201 | ko04626 |
| 5 | [Endocytosis](file:///C:\Documents%20and%20Settings\Administrator\%25E6%25A1%258C%25E9%259D%25A2\%25E9%2599%2588%25E5%25BB%25BA%25E8%258D%25A3\%25E5%259F%25BA%25E5%259B%25A0%25E7%25BB%2584%25E6%2595%25B0%25E6%258D%25AE\%25E5%259F%25BA%25E5%259B%25A0%25E7%25BB%2584%25E6%2595%25B0%25E6%258D%25AE\HSZP10089\annotation\KEGG\All-Unigene.fa.htm#gene5) | 1104 | ko04144 |
| 6 | [Glycerophospholipid metabolism](file:///C:\Documents%20and%20Settings\Administrator\%25E6%25A1%258C%25E9%259D%25A2\%25E9%2599%2588%25E5%25BB%25BA%25E8%258D%25A3\%25E5%259F%25BA%25E5%259B%25A0%25E7%25BB%2584%25E6%2595%25B0%25E6%258D%25AE\%25E5%259F%25BA%25E5%259B%25A0%25E7%25BB%2584%25E6%2595%25B0%25E6%258D%25AE\HSZP10089\annotation\KEGG\All-Unigene.fa.htm#gene6) | 1047 | ko00564 |
| 7 | [Plant hormone signal transduction](file:///C:\Documents%20and%20Settings\Administrator\%25E6%25A1%258C%25E9%259D%25A2\%25E9%2599%2588%25E5%25BB%25BA%25E8%258D%25A3\%25E5%259F%25BA%25E5%259B%25A0%25E7%25BB%2584%25E6%2595%25B0%25E6%258D%25AE\%25E5%259F%25BA%25E5%259B%25A0%25E7%25BB%2584%25E6%2595%25B0%25E6%258D%25AE\HSZP10089\annotation\KEGG\All-Unigene.fa.htm#gene7) | 973 | ko04075 |
| 8 | [Ether lipid metabolism](file:///C:\Documents%20and%20Settings\Administrator\%25E6%25A1%258C%25E9%259D%25A2\%25E9%2599%2588%25E5%25BB%25BA%25E8%258D%25A3\%25E5%259F%25BA%25E5%259B%25A0%25E7%25BB%2584%25E6%2595%25B0%25E6%258D%25AE\%25E5%259F%25BA%25E5%259B%25A0%25E7%25BB%2584%25E6%2595%25B0%25E6%258D%25AE\HSZP10089\annotation\KEGG\All-Unigene.fa.htm#gene8) | 954 | ko00565 |
| 9 | [mRNA surveillance pathway](file:///C:\Documents%20and%20Settings\Administrator\%25E6%25A1%258C%25E9%259D%25A2\%25E9%2599%2588%25E5%25BB%25BA%25E8%258D%25A3\%25E5%259F%25BA%25E5%259B%25A0%25E7%25BB%2584%25E6%2595%25B0%25E6%258D%25AE\%25E5%259F%25BA%25E5%259B%25A0%25E7%25BB%2584%25E6%2595%25B0%25E6%258D%25AE\HSZP10089\annotation\KEGG\All-Unigene.fa.htm#gene9) | 864 | ko03015 |
| 10 | [Spliceosome](file:///C:\Documents%20and%20Settings\Administrator\%25E6%25A1%258C%25E9%259D%25A2\%25E9%2599%2588%25E5%25BB%25BA%25E8%258D%25A3\%25E5%259F%25BA%25E5%259B%25A0%25E7%25BB%2584%25E6%2595%25B0%25E6%258D%25AE\%25E5%259F%25BA%25E5%259B%25A0%25E7%25BB%2584%25E6%2595%25B0%25E6%258D%25AE\HSZP10089\annotation\KEGG\All-Unigene.fa.htm#gene10) | 805 | ko03040 |
| 11 | [Ribosome](file:///C:\Documents%20and%20Settings\Administrator\%25E6%25A1%258C%25E9%259D%25A2\%25E9%2599%2588%25E5%25BB%25BA%25E8%258D%25A3\%25E5%259F%25BA%25E5%259B%25A0%25E7%25BB%2584%25E6%2595%25B0%25E6%258D%25AE\%25E5%259F%25BA%25E5%259B%25A0%25E7%25BB%2584%25E6%2595%25B0%25E6%258D%25AE\HSZP10089\annotation\KEGG\All-Unigene.fa.htm#gene11) | 714 | ko03010 |
| 12 | [Protein processing in endoplasmic reticulum](file:///C:\Documents%20and%20Settings\Administrator\%25E6%25A1%258C%25E9%259D%25A2\%25E9%2599%2588%25E5%25BB%25BA%25E8%258D%25A3\%25E5%259F%25BA%25E5%259B%25A0%25E7%25BB%2584%25E6%2595%25B0%25E6%258D%25AE\%25E5%259F%25BA%25E5%259B%25A0%25E7%25BB%2584%25E6%2595%25B0%25E6%258D%25AE\HSZP10089\annotation\KEGG\All-Unigene.fa.htm#gene12) | 529 | ko04141 |
| 13 | [Purine metabolism](file:///C:\Documents%20and%20Settings\Administrator\%25E6%25A1%258C%25E9%259D%25A2\%25E9%2599%2588%25E5%25BB%25BA%25E8%258D%25A3\%25E5%259F%25BA%25E5%259B%25A0%25E7%25BB%2584%25E6%2595%25B0%25E6%258D%25AE\%25E5%259F%25BA%25E5%259B%25A0%25E7%25BB%2584%25E6%2595%25B0%25E6%258D%25AE\HSZP10089\annotation\KEGG\All-Unigene.fa.htm#gene13) | 493 | ko00230 |
| 14 | [Pyrimidine metabolism](file:///C:\Documents%20and%20Settings\Administrator\%25E6%25A1%258C%25E9%259D%25A2\%25E9%2599%2588%25E5%25BB%25BA%25E8%258D%25A3\%25E5%259F%25BA%25E5%259B%25A0%25E7%25BB%2584%25E6%2595%25B0%25E6%258D%25AE\%25E5%259F%25BA%25E5%259B%25A0%25E7%25BB%2584%25E6%2595%25B0%25E6%258D%25AE\HSZP10089\annotation\KEGG\All-Unigene.fa.htm#gene14) | 425 | ko00240 |
| 15 | [Oxidative phosphorylation](file:///C:\Documents%20and%20Settings\Administrator\%25E6%25A1%258C%25E9%259D%25A2\%25E9%2599%2588%25E5%25BB%25BA%25E8%258D%25A3\%25E5%259F%25BA%25E5%259B%25A0%25E7%25BB%2584%25E6%2595%25B0%25E6%258D%25AE\%25E5%259F%25BA%25E5%259B%25A0%25E7%25BB%2584%25E6%2595%25B0%25E6%258D%25AE\HSZP10089\annotation\KEGG\All-Unigene.fa.htm#gene15) | 365 | ko00190 |
| 16 | [Phenylpropanoid biosynthesis](file:///C:\Documents%20and%20Settings\Administrator\%25E6%25A1%258C%25E9%259D%25A2\%25E9%2599%2588%25E5%25BB%25BA%25E8%258D%25A3\%25E5%259F%25BA%25E5%259B%25A0%25E7%25BB%2584%25E6%2595%25B0%25E6%258D%25AE\%25E5%259F%25BA%25E5%259B%25A0%25E7%25BB%2584%25E6%2595%25B0%25E6%258D%25AE\HSZP10089\annotation\KEGG\All-Unigene.fa.htm#gene16) | 365 | ko00940 |
| 17 | [Glycolysis / Gluconeogenesis](file:///C:\Documents%20and%20Settings\Administrator\%25E6%25A1%258C%25E9%259D%25A2\%25E9%2599%2588%25E5%25BB%25BA%25E8%258D%25A3\%25E5%259F%25BA%25E5%259B%25A0%25E7%25BB%2584%25E6%2595%25B0%25E6%258D%25AE\%25E5%259F%25BA%25E5%259B%25A0%25E7%25BB%2584%25E6%2595%25B0%25E6%258D%25AE\HSZP10089\annotation\KEGG\All-Unigene.fa.htm#gene17) | 354 | ko00010 |
| 18 | [Ribosome biogenesis in eukaryotes](file:///C:\Documents%20and%20Settings\Administrator\%25E6%25A1%258C%25E9%259D%25A2\%25E9%2599%2588%25E5%25BB%25BA%25E8%258D%25A3\%25E5%259F%25BA%25E5%259B%25A0%25E7%25BB%2584%25E6%2595%25B0%25E6%258D%25AE\%25E5%259F%25BA%25E5%259B%25A0%25E7%25BB%2584%25E6%2595%25B0%25E6%258D%25AE\HSZP10089\annotation\KEGG\All-Unigene.fa.htm#gene18) | 341 | ko03008 |
| 19 | [Starch and sucrose metabolism](file:///C:\Documents%20and%20Settings\Administrator\%25E6%25A1%258C%25E9%259D%25A2\%25E9%2599%2588%25E5%25BB%25BA%25E8%258D%25A3\%25E5%259F%25BA%25E5%259B%25A0%25E7%25BB%2584%25E6%2595%25B0%25E6%258D%25AE\%25E5%259F%25BA%25E5%259B%25A0%25E7%25BB%2584%25E6%2595%25B0%25E6%258D%25AE\HSZP10089\annotation\KEGG\All-Unigene.fa.htm#gene19) | 318 | ko00500 |
| 20 | [Ubiquitin mediated proteolysis](file:///C:\Documents%20and%20Settings\Administrator\%25E6%25A1%258C%25E9%259D%25A2\%25E9%2599%2588%25E5%25BB%25BA%25E8%258D%25A3\%25E5%259F%25BA%25E5%259B%25A0%25E7%25BB%2584%25E6%2595%25B0%25E6%258D%25AE\%25E5%259F%25BA%25E5%259B%25A0%25E7%25BB%2584%25E6%2595%25B0%25E6%258D%25AE\HSZP10089\annotation\KEGG\All-Unigene.fa.htm#gene20) | 304 | ko04120 |
| 21 | [RNA degradation](file:///C:\Documents%20and%20Settings\Administrator\%25E6%25A1%258C%25E9%259D%25A2\%25E9%2599%2588%25E5%25BB%25BA%25E8%258D%25A3\%25E5%259F%25BA%25E5%259B%25A0%25E7%25BB%2584%25E6%2595%25B0%25E6%258D%25AE\%25E5%259F%25BA%25E5%259B%25A0%25E7%25BB%2584%25E6%2595%25B0%25E6%258D%25AE\HSZP10089\annotation\KEGG\All-Unigene.fa.htm#gene21) | 296 | ko03018 |
| 22 | [RNA polymerase](file:///C:\Documents%20and%20Settings\Administrator\%25E6%25A1%258C%25E9%259D%25A2\%25E9%2599%2588%25E5%25BB%25BA%25E8%258D%25A3\%25E5%259F%25BA%25E5%259B%25A0%25E7%25BB%2584%25E6%2595%25B0%25E6%258D%25AE\%25E5%259F%25BA%25E5%259B%25A0%25E7%25BB%2584%25E6%2595%25B0%25E6%258D%25AE\HSZP10089\annotation\KEGG\All-Unigene.fa.htm#gene22) | 275 | ko03020 |
| 23 | [Amino sugar and nucleotide sugar metabolism](file:///C:\Documents%20and%20Settings\Administrator\%25E6%25A1%258C%25E9%259D%25A2\%25E9%2599%2588%25E5%25BB%25BA%25E8%258D%25A3\%25E5%259F%25BA%25E5%259B%25A0%25E7%25BB%2584%25E6%2595%25B0%25E6%258D%25AE\%25E5%259F%25BA%25E5%259B%25A0%25E7%25BB%2584%25E6%2595%25B0%25E6%258D%25AE\HSZP10089\annotation\KEGG\All-Unigene.fa.htm#gene23) | 232 | ko00520 |
| 24 | [Flavonoid biosynthesis](file:///C:\Documents%20and%20Settings\Administrator\%25E6%25A1%258C%25E9%259D%25A2\%25E9%2599%2588%25E5%25BB%25BA%25E8%258D%25A3\%25E5%259F%25BA%25E5%259B%25A0%25E7%25BB%2584%25E6%2595%25B0%25E6%258D%25AE\%25E5%259F%25BA%25E5%259B%25A0%25E7%25BB%2584%25E6%2595%25B0%25E6%258D%25AE\HSZP10089\annotation\KEGG\All-Unigene.fa.htm#gene24) | 228 | ko00941 |
| 25 | [Phagosome](file:///C:\Documents%20and%20Settings\Administrator\%25E6%25A1%258C%25E9%259D%25A2\%25E9%2599%2588%25E5%25BB%25BA%25E8%258D%25A3\%25E5%259F%25BA%25E5%259B%25A0%25E7%25BB%2584%25E6%2595%25B0%25E6%258D%25AE\%25E5%259F%25BA%25E5%259B%25A0%25E7%25BB%2584%25E6%2595%25B0%25E6%258D%25AE\HSZP10089\annotation\KEGG\All-Unigene.fa.htm#gene25) | 225 | ko04145 |
| 26 | [Pyruvate metabolism](file:///C:\Documents%20and%20Settings\Administrator\%25E6%25A1%258C%25E9%259D%25A2\%25E9%2599%2588%25E5%25BB%25BA%25E8%258D%25A3\%25E5%259F%25BA%25E5%259B%25A0%25E7%25BB%2584%25E6%2595%25B0%25E6%258D%25AE\%25E5%259F%25BA%25E5%259B%25A0%25E7%25BB%2584%25E6%2595%25B0%25E6%258D%25AE\HSZP10089\annotation\KEGG\All-Unigene.fa.htm#gene26) | 225 | ko00620 |
| 27 | [Peroxisome](file:///C:\Documents%20and%20Settings\Administrator\%25E6%25A1%258C%25E9%259D%25A2\%25E9%2599%2588%25E5%25BB%25BA%25E8%258D%25A3\%25E5%259F%25BA%25E5%259B%25A0%25E7%25BB%2584%25E6%2595%25B0%25E6%258D%25AE\%25E5%259F%25BA%25E5%259B%25A0%25E7%25BB%2584%25E6%2595%25B0%25E6%258D%25AE\HSZP10089\annotation\KEGG\All-Unigene.fa.htm#gene27) | 221 | ko04146 |
| 28 | [Stilbenoid, diarylheptanoid and gingerol biosynthesis](file:///C:\Documents%20and%20Settings\Administrator\%25E6%25A1%258C%25E9%259D%25A2\%25E9%2599%2588%25E5%25BB%25BA%25E8%258D%25A3\%25E5%259F%25BA%25E5%259B%25A0%25E7%25BB%2584%25E6%2595%25B0%25E6%258D%25AE\%25E5%259F%25BA%25E5%259B%25A0%25E7%25BB%2584%25E6%2595%25B0%25E6%258D%25AE\HSZP10089\annotation\KEGG\All-Unigene.fa.htm#gene28) | 204 | ko00945 |
| 29 | [Limonene and pinene degradation](file:///C:\Documents%20and%20Settings\Administrator\%25E6%25A1%258C%25E9%259D%25A2\%25E9%2599%2588%25E5%25BB%25BA%25E8%258D%25A3\%25E5%259F%25BA%25E5%259B%25A0%25E7%25BB%2584%25E6%2595%25B0%25E6%258D%25AE\%25E5%259F%25BA%25E5%259B%25A0%25E7%25BB%2584%25E6%2595%25B0%25E6%258D%25AE\HSZP10089\annotation\KEGG\All-Unigene.fa.htm#gene29) | 203 | ko00903 |
| 30 | [Pentose phosphate pathway](file:///C:\Documents%20and%20Settings\Administrator\%25E6%25A1%258C%25E9%259D%25A2\%25E9%2599%2588%25E5%25BB%25BA%25E8%258D%25A3\%25E5%259F%25BA%25E5%259B%25A0%25E7%25BB%2584%25E6%2595%25B0%25E6%258D%25AE\%25E5%259F%25BA%25E5%259B%25A0%25E7%25BB%2584%25E6%2595%25B0%25E6%258D%25AE\HSZP10089\annotation\KEGG\All-Unigene.fa.htm#gene30) | 189 | ko00030 |
| 31 | [Circadian rhythm - plant](file:///C:\Documents%20and%20Settings\Administrator\%25E6%25A1%258C%25E9%259D%25A2\%25E9%2599%2588%25E5%25BB%25BA%25E8%258D%25A3\%25E5%259F%25BA%25E5%259B%25A0%25E7%25BB%2584%25E6%2595%25B0%25E6%258D%25AE\%25E5%259F%25BA%25E5%259B%25A0%25E7%25BB%2584%25E6%2595%25B0%25E6%258D%25AE\HSZP10089\annotation\KEGG\All-Unigene.fa.htm#gene31) | 174 | ko04712 |
| 32 | [Arginine and proline metabolism](file:///C:\Documents%20and%20Settings\Administrator\%25E6%25A1%258C%25E9%259D%25A2\%25E9%2599%2588%25E5%25BB%25BA%25E8%258D%25A3\%25E5%259F%25BA%25E5%259B%25A0%25E7%25BB%2584%25E6%2595%25B0%25E6%258D%25AE\%25E5%259F%25BA%25E5%259B%25A0%25E7%25BB%2584%25E6%2595%25B0%25E6%258D%25AE\HSZP10089\annotation\KEGG\All-Unigene.fa.htm#gene32) | 172 | ko00330 |
| 33 | [Galactose metabolism](file:///C:\Documents%20and%20Settings\Administrator\%25E6%25A1%258C%25E9%259D%25A2\%25E9%2599%2588%25E5%25BB%25BA%25E8%258D%25A3\%25E5%259F%25BA%25E5%259B%25A0%25E7%25BB%2584%25E6%2595%25B0%25E6%258D%25AE\%25E5%259F%25BA%25E5%259B%25A0%25E7%25BB%2584%25E6%2595%25B0%25E6%258D%25AE\HSZP10089\annotation\KEGG\All-Unigene.fa.htm#gene33) | 171 | ko00052 |
| 34 | [Fructose and mannose metabolism](file:///C:\Documents%20and%20Settings\Administrator\%25E6%25A1%258C%25E9%259D%25A2\%25E9%2599%2588%25E5%25BB%25BA%25E8%258D%25A3\%25E5%259F%25BA%25E5%259B%25A0%25E7%25BB%2584%25E6%2595%25B0%25E6%258D%25AE\%25E5%259F%25BA%25E5%259B%25A0%25E7%25BB%2584%25E6%2595%25B0%25E6%258D%25AE\HSZP10089\annotation\KEGG\All-Unigene.fa.htm#gene34) | 169 | ko00051 |
| 35 | [Cysteine and methionine metabolism](file:///C:\Documents%20and%20Settings\Administrator\%25E6%25A1%258C%25E9%259D%25A2\%25E9%2599%2588%25E5%25BB%25BA%25E8%258D%25A3\%25E5%259F%25BA%25E5%259B%25A0%25E7%25BB%2584%25E6%2595%25B0%25E6%258D%25AE\%25E5%259F%25BA%25E5%259B%25A0%25E7%25BB%2584%25E6%2595%25B0%25E6%258D%25AE\HSZP10089\annotation\KEGG\All-Unigene.fa.htm#gene35) | 168 | ko00270 |
| 36 | [Carbon fixation in photosynthetic organisms](file:///C:\Documents%20and%20Settings\Administrator\%25E6%25A1%258C%25E9%259D%25A2\%25E9%2599%2588%25E5%25BB%25BA%25E8%258D%25A3\%25E5%259F%25BA%25E5%259B%25A0%25E7%25BB%2584%25E6%2595%25B0%25E6%258D%25AE\%25E5%259F%25BA%25E5%259B%25A0%25E7%25BB%2584%25E6%2595%25B0%25E6%258D%25AE\HSZP10089\annotation\KEGG\All-Unigene.fa.htm#gene36) | 166 | ko00710 |
| 37 | [Zeatin biosynthesis](file:///C:\Documents%20and%20Settings\Administrator\%25E6%25A1%258C%25E9%259D%25A2\%25E9%2599%2588%25E5%25BB%25BA%25E8%258D%25A3\%25E5%259F%25BA%25E5%259B%25A0%25E7%25BB%2584%25E6%2595%25B0%25E6%258D%25AE\%25E5%259F%25BA%25E5%259B%25A0%25E7%25BB%2584%25E6%2595%25B0%25E6%258D%25AE\HSZP10089\annotation\KEGG\All-Unigene.fa.htm#gene37) | 165 | ko00908 |
| 38 | [Fatty acid metabolism](file:///C:\Documents%20and%20Settings\Administrator\%25E6%25A1%258C%25E9%259D%25A2\%25E9%2599%2588%25E5%25BB%25BA%25E8%258D%25A3\%25E5%259F%25BA%25E5%259B%25A0%25E7%25BB%2584%25E6%2595%25B0%25E6%258D%25AE\%25E5%259F%25BA%25E5%259B%25A0%25E7%25BB%2584%25E6%2595%25B0%25E6%258D%25AE\HSZP10089\annotation\KEGG\All-Unigene.fa.htm#gene38) | 163 | ko00071 |
| 39 | [Glutathione metabolism](file:///C:\Documents%20and%20Settings\Administrator\%25E6%25A1%258C%25E9%259D%25A2\%25E9%2599%2588%25E5%25BB%25BA%25E8%258D%25A3\%25E5%259F%25BA%25E5%259B%25A0%25E7%25BB%2584%25E6%2595%25B0%25E6%258D%25AE\%25E5%259F%25BA%25E5%259B%25A0%25E7%25BB%2584%25E6%2595%25B0%25E6%258D%25AE\HSZP10089\annotation\KEGG\All-Unigene.fa.htm#gene39) | 162 | ko00480 |
| 40 | [Phenylalanine metabolism](file:///C:\Documents%20and%20Settings\Administrator\%25E6%25A1%258C%25E9%259D%25A2\%25E9%2599%2588%25E5%25BB%25BA%25E8%258D%25A3\%25E5%259F%25BA%25E5%259B%25A0%25E7%25BB%2584%25E6%2595%25B0%25E6%258D%25AE\%25E5%259F%25BA%25E5%259B%25A0%25E7%25BB%2584%25E6%2595%25B0%25E6%258D%25AE\HSZP10089\annotation\KEGG\All-Unigene.fa.htm#gene40) | 158 | ko00360 |
| 41 | [Pentose and glucuronate interconversions](file:///C:\Documents%20and%20Settings\Administrator\%25E6%25A1%258C%25E9%259D%25A2\%25E9%2599%2588%25E5%25BB%25BA%25E8%258D%25A3\%25E5%259F%25BA%25E5%259B%25A0%25E7%25BB%2584%25E6%2595%25B0%25E6%258D%25AE\%25E5%259F%25BA%25E5%259B%25A0%25E7%25BB%2584%25E6%2595%25B0%25E6%258D%25AE\HSZP10089\annotation\KEGG\All-Unigene.fa.htm#gene41) | 148 | ko00040 |
| 42 | [Nucleotide excision repair](file:///C:\Documents%20and%20Settings\Administrator\%25E6%25A1%258C%25E9%259D%25A2\%25E9%2599%2588%25E5%25BB%25BA%25E8%258D%25A3\%25E5%259F%25BA%25E5%259B%25A0%25E7%25BB%2584%25E6%2595%25B0%25E6%258D%25AE\%25E5%259F%25BA%25E5%259B%25A0%25E7%25BB%2584%25E6%2595%25B0%25E6%258D%25AE\HSZP10089\annotation\KEGG\All-Unigene.fa.htm#gene42) | 145 | ko03420 |
| 43 | [Glycerolipid metabolism](file:///C:\Documents%20and%20Settings\Administrator\%25E6%25A1%258C%25E9%259D%25A2\%25E9%2599%2588%25E5%25BB%25BA%25E8%258D%25A3\%25E5%259F%25BA%25E5%259B%25A0%25E7%25BB%2584%25E6%2595%25B0%25E6%258D%25AE\%25E5%259F%25BA%25E5%259B%25A0%25E7%25BB%2584%25E6%2595%25B0%25E6%258D%25AE\HSZP10089\annotation\KEGG\All-Unigene.fa.htm#gene43) | 143 | ko00561 |
| 44 | [Basal transcription factors](file:///C:\Documents%20and%20Settings\Administrator\%25E6%25A1%258C%25E9%259D%25A2\%25E9%2599%2588%25E5%25BB%25BA%25E8%258D%25A3\%25E5%259F%25BA%25E5%259B%25A0%25E7%25BB%2584%25E6%2595%25B0%25E6%258D%25AE\%25E5%259F%25BA%25E5%259B%25A0%25E7%25BB%2584%25E6%2595%25B0%25E6%258D%25AE\HSZP10089\annotation\KEGG\All-Unigene.fa.htm#gene44) | 142 | ko03022 |
| 45 | [Ascorbate and aldarate metabolism](file:///C:\Documents%20and%20Settings\Administrator\%25E6%25A1%258C%25E9%259D%25A2\%25E9%2599%2588%25E5%25BB%25BA%25E8%258D%25A3\%25E5%259F%25BA%25E5%259B%25A0%25E7%25BB%2584%25E6%2595%25B0%25E6%258D%25AE\%25E5%259F%25BA%25E5%259B%25A0%25E7%25BB%2584%25E6%2595%25B0%25E6%258D%25AE\HSZP10089\annotation\KEGG\All-Unigene.fa.htm#gene45) | 142 | ko00053 |
| 46 | [Tyrosine metabolism](file:///C:\Documents%20and%20Settings\Administrator\%25E6%25A1%258C%25E9%259D%25A2\%25E9%2599%2588%25E5%25BB%25BA%25E8%258D%25A3\%25E5%259F%25BA%25E5%259B%25A0%25E7%25BB%2584%25E6%2595%25B0%25E6%258D%25AE\%25E5%259F%25BA%25E5%259B%25A0%25E7%25BB%2584%25E6%2595%25B0%25E6%258D%25AE\HSZP10089\annotation\KEGG\All-Unigene.fa.htm#gene46) | 130 | ko00350 |
| 47 | [Citrate cycle (TCA cycle)](file:///C:\Documents%20and%20Settings\Administrator\%25E6%25A1%258C%25E9%259D%25A2\%25E9%2599%2588%25E5%25BB%25BA%25E8%258D%25A3\%25E5%259F%25BA%25E5%259B%25A0%25E7%25BB%2584%25E6%2595%25B0%25E6%258D%25AE\%25E5%259F%25BA%25E5%259B%25A0%25E7%25BB%2584%25E6%2595%25B0%25E6%258D%25AE\HSZP10089\annotation\KEGG\All-Unigene.fa.htm#gene47) | 128 | ko00020 |
| 48 | [Propanoate metabolism](file:///C:\Documents%20and%20Settings\Administrator\%25E6%25A1%258C%25E9%259D%25A2\%25E9%2599%2588%25E5%25BB%25BA%25E8%258D%25A3\%25E5%259F%25BA%25E5%259B%25A0%25E7%25BB%2584%25E6%2595%25B0%25E6%258D%25AE\%25E5%259F%25BA%25E5%259B%25A0%25E7%25BB%2584%25E6%2595%25B0%25E6%258D%25AE\HSZP10089\annotation\KEGG\All-Unigene.fa.htm#gene48) | 127 | ko00640 |
| 49 | [ABC transporters](file:///C:\Documents%20and%20Settings\Administrator\%25E6%25A1%258C%25E9%259D%25A2\%25E9%2599%2588%25E5%25BB%25BA%25E8%258D%25A3\%25E5%259F%25BA%25E5%259B%25A0%25E7%25BB%2584%25E6%2595%25B0%25E6%258D%25AE\%25E5%259F%25BA%25E5%259B%25A0%25E7%25BB%2584%25E6%2595%25B0%25E6%258D%25AE\HSZP10089\annotation\KEGG\All-Unigene.fa.htm#gene49) | 125 | ko02010 |
| 50 | [Inositol phosphate metabolism](file:///C:\Documents%20and%20Settings\Administrator\%25E6%25A1%258C%25E9%259D%25A2\%25E9%2599%2588%25E5%25BB%25BA%25E8%258D%25A3\%25E5%259F%25BA%25E5%259B%25A0%25E7%25BB%2584%25E6%2595%25B0%25E6%258D%25AE\%25E5%259F%25BA%25E5%259B%25A0%25E7%25BB%2584%25E6%2595%25B0%25E6%258D%25AE\HSZP10089\annotation\KEGG\All-Unigene.fa.htm#gene50) | 124 | ko00562 |
| 51 | [Valine, leucine and isoleucine degradation](file:///C:\Documents%20and%20Settings\Administrator\%25E6%25A1%258C%25E9%259D%25A2\%25E9%2599%2588%25E5%25BB%25BA%25E8%258D%25A3\%25E5%259F%25BA%25E5%259B%25A0%25E7%25BB%2584%25E6%2595%25B0%25E6%258D%25AE\%25E5%259F%25BA%25E5%259B%25A0%25E7%25BB%2584%25E6%2595%25B0%25E6%258D%25AE\HSZP10089\annotation\KEGG\All-Unigene.fa.htm#gene51) | 123 | ko00280 |
| 52 | [Cyanoamino acid metabolism](file:///C:\Documents%20and%20Settings\Administrator\%25E6%25A1%258C%25E9%259D%25A2\%25E9%2599%2588%25E5%25BB%25BA%25E8%258D%25A3\%25E5%259F%25BA%25E5%259B%25A0%25E7%25BB%2584%25E6%2595%25B0%25E6%258D%25AE\%25E5%259F%25BA%25E5%259B%25A0%25E7%25BB%2584%25E6%2595%25B0%25E6%258D%25AE\HSZP10089\annotation\KEGG\All-Unigene.fa.htm#gene52) | 123 | ko00460 |
| 53 | [Glyoxylate and dicarboxylate metabolism](file:///C:\Documents%20and%20Settings\Administrator\%25E6%25A1%258C%25E9%259D%25A2\%25E9%2599%2588%25E5%25BB%25BA%25E8%258D%25A3\%25E5%259F%25BA%25E5%259B%25A0%25E7%25BB%2584%25E6%2595%25B0%25E6%258D%25AE\%25E5%259F%25BA%25E5%259B%25A0%25E7%25BB%2584%25E6%2595%25B0%25E6%258D%25AE\HSZP10089\annotation\KEGG\All-Unigene.fa.htm#gene53) | 122 | ko00630 |
| 54 | [alpha-Linolenic acid metabolism](file:///C:\Documents%20and%20Settings\Administrator\%25E6%25A1%258C%25E9%259D%25A2\%25E9%2599%2588%25E5%25BB%25BA%25E8%258D%25A3\%25E5%259F%25BA%25E5%259B%25A0%25E7%25BB%2584%25E6%2595%25B0%25E6%258D%25AE\%25E5%259F%25BA%25E5%259B%25A0%25E7%25BB%2584%25E6%2595%25B0%25E6%258D%25AE\HSZP10089\annotation\KEGG\All-Unigene.fa.htm#gene54) | 119 | ko00592 |
| 55 | [Tryptophan metabolism](file:///C:\Documents%20and%20Settings\Administrator\%25E6%25A1%258C%25E9%259D%25A2\%25E9%2599%2588%25E5%25BB%25BA%25E8%258D%25A3\%25E5%259F%25BA%25E5%259B%25A0%25E7%25BB%2584%25E6%2595%25B0%25E6%258D%25AE\%25E5%259F%25BA%25E5%259B%25A0%25E7%25BB%2584%25E6%2595%25B0%25E6%258D%25AE\HSZP10089\annotation\KEGG\All-Unigene.fa.htm#gene55) | 119 | ko00380 |
| 56 | [Phosphatidylinositol signaling system](file:///C:\Documents%20and%20Settings\Administrator\%25E6%25A1%258C%25E9%259D%25A2\%25E9%2599%2588%25E5%25BB%25BA%25E8%258D%25A3\%25E5%259F%25BA%25E5%259B%25A0%25E7%25BB%2584%25E6%2595%25B0%25E6%258D%25AE\%25E5%259F%25BA%25E5%259B%25A0%25E7%25BB%2584%25E6%2595%25B0%25E6%258D%25AE\HSZP10089\annotation\KEGG\All-Unigene.fa.htm#gene56) | 119 | ko04070 |
| 57 | [beta-Alanine metabolism](file:///C:\Documents%20and%20Settings\Administrator\%25E6%25A1%258C%25E9%259D%25A2\%25E9%2599%2588%25E5%25BB%25BA%25E8%258D%25A3\%25E5%259F%25BA%25E5%259B%25A0%25E7%25BB%2584%25E6%2595%25B0%25E6%258D%25AE\%25E5%259F%25BA%25E5%259B%25A0%25E7%25BB%2584%25E6%2595%25B0%25E6%258D%25AE\HSZP10089\annotation\KEGG\All-Unigene.fa.htm#gene57) | 117 | ko00410 |
| 58 | [Alanine, aspartate and glutamate metabolism](file:///C:\Documents%20and%20Settings\Administrator\%25E6%25A1%258C%25E9%259D%25A2\%25E9%2599%2588%25E5%25BB%25BA%25E8%258D%25A3\%25E5%259F%25BA%25E5%259B%25A0%25E7%25BB%2584%25E6%2595%25B0%25E6%258D%25AE\%25E5%259F%25BA%25E5%259B%25A0%25E7%25BB%2584%25E6%2595%25B0%25E6%258D%25AE\HSZP10089\annotation\KEGG\All-Unigene.fa.htm#gene58) | 113 | ko00250 |
| 59 | [Carotenoid biosynthesis](file:///C:\Documents%20and%20Settings\Administrator\%25E6%25A1%258C%25E9%259D%25A2\%25E9%2599%2588%25E5%25BB%25BA%25E8%258D%25A3\%25E5%259F%25BA%25E5%259B%25A0%25E7%25BB%2584%25E6%2595%25B0%25E6%258D%25AE\%25E5%259F%25BA%25E5%259B%25A0%25E7%25BB%2584%25E6%2595%25B0%25E6%258D%25AE\HSZP10089\annotation\KEGG\All-Unigene.fa.htm#gene59) | 108 | ko00906 |
| 60 | [Biosynthesis of unsaturated fatty acids](file:///C:\Documents%20and%20Settings\Administrator\%25E6%25A1%258C%25E9%259D%25A2\%25E9%2599%2588%25E5%25BB%25BA%25E8%258D%25A3\%25E5%259F%25BA%25E5%259B%25A0%25E7%25BB%2584%25E6%2595%25B0%25E6%258D%25AE\%25E5%259F%25BA%25E5%259B%25A0%25E7%25BB%2584%25E6%2595%25B0%25E6%258D%25AE\HSZP10089\annotation\KEGG\All-Unigene.fa.htm#gene60) | 107 | ko01040 |
| 61 | [Regulation of autophagy](file:///C:\Documents%20and%20Settings\Administrator\%25E6%25A1%258C%25E9%259D%25A2\%25E9%2599%2588%25E5%25BB%25BA%25E8%258D%25A3\%25E5%259F%25BA%25E5%259B%25A0%25E7%25BB%2584%25E6%2595%25B0%25E6%258D%25AE\%25E5%259F%25BA%25E5%259B%25A0%25E7%25BB%2584%25E6%2595%25B0%25E6%258D%25AE\HSZP10089\annotation\KEGG\All-Unigene.fa.htm#gene61) | 107 | ko04140 |
| 62 | [Aminoacyl-tRNA biosynthesis](file:///C:\Documents%20and%20Settings\Administrator\%25E6%25A1%258C%25E9%259D%25A2\%25E9%2599%2588%25E5%25BB%25BA%25E8%258D%25A3\%25E5%259F%25BA%25E5%259B%25A0%25E7%25BB%2584%25E6%2595%25B0%25E6%258D%25AE\%25E5%259F%25BA%25E5%259B%25A0%25E7%25BB%2584%25E6%2595%25B0%25E6%258D%25AE\HSZP10089\annotation\KEGG\All-Unigene.fa.htm#gene62) | 106 | ko00970 |
| 63 | [Homologous recombination](file:///C:\Documents%20and%20Settings\Administrator\%25E6%25A1%258C%25E9%259D%25A2\%25E9%2599%2588%25E5%25BB%25BA%25E8%258D%25A3\%25E5%259F%25BA%25E5%259B%25A0%25E7%25BB%2584%25E6%2595%25B0%25E6%258D%25AE\%25E5%259F%25BA%25E5%259B%25A0%25E7%25BB%2584%25E6%2595%25B0%25E6%258D%25AE\HSZP10089\annotation\KEGG\All-Unigene.fa.htm#gene63) | 103 | ko03440 |
| 64 | [Porphyrin and chlorophyll metabolism](file:///C:\Documents%20and%20Settings\Administrator\%25E6%25A1%258C%25E9%259D%25A2\%25E9%2599%2588%25E5%25BB%25BA%25E8%258D%25A3\%25E5%259F%25BA%25E5%259B%25A0%25E7%25BB%2584%25E6%2595%25B0%25E6%258D%25AE\%25E5%259F%25BA%25E5%259B%25A0%25E7%25BB%2584%25E6%2595%25B0%25E6%258D%25AE\HSZP10089\annotation\KEGG\All-Unigene.fa.htm#gene64) | 101 | ko00860 |
| 65 | [Lysine degradation](file:///C:\Documents%20and%20Settings\Administrator\%25E6%25A1%258C%25E9%259D%25A2\%25E9%2599%2588%25E5%25BB%25BA%25E8%258D%25A3\%25E5%259F%25BA%25E5%259B%25A0%25E7%25BB%2584%25E6%2595%25B0%25E6%258D%25AE\%25E5%259F%25BA%25E5%259B%25A0%25E7%25BB%2584%25E6%2595%25B0%25E6%258D%25AE\HSZP10089\annotation\KEGG\All-Unigene.fa.htm#gene65) | 95 | ko00310 |
| 66 | [Base excision repair](file:///C:\Documents%20and%20Settings\Administrator\%25E6%25A1%258C%25E9%259D%25A2\%25E9%2599%2588%25E5%25BB%25BA%25E8%258D%25A3\%25E5%259F%25BA%25E5%259B%25A0%25E7%25BB%2584%25E6%2595%25B0%25E6%258D%25AE\%25E5%259F%25BA%25E5%259B%25A0%25E7%25BB%2584%25E6%2595%25B0%25E6%258D%25AE\HSZP10089\annotation\KEGG\All-Unigene.fa.htm#gene66) | 95 | ko03410 |
| 67 | [Glycine, serine and threonine metabolism](file:///C:\Documents%20and%20Settings\Administrator\%25E6%25A1%258C%25E9%259D%25A2\%25E9%2599%2588%25E5%25BB%25BA%25E8%258D%25A3\%25E5%259F%25BA%25E5%259B%25A0%25E7%25BB%2584%25E6%2595%25B0%25E6%258D%25AE\%25E5%259F%25BA%25E5%259B%25A0%25E7%25BB%2584%25E6%2595%25B0%25E6%258D%25AE\HSZP10089\annotation\KEGG\All-Unigene.fa.htm#gene67) | 95 | ko00260 |
| 68 | [Photosynthesis](file:///C:\Documents%20and%20Settings\Administrator\%25E6%25A1%258C%25E9%259D%25A2\%25E9%2599%2588%25E5%25BB%25BA%25E8%258D%25A3\%25E5%259F%25BA%25E5%259B%25A0%25E7%25BB%2584%25E6%2595%25B0%25E6%258D%25AE\%25E5%259F%25BA%25E5%259B%25A0%25E7%25BB%2584%25E6%2595%25B0%25E6%258D%25AE\HSZP10089\annotation\KEGG\All-Unigene.fa.htm#gene68) | 93 | ko00195 |
| 69 | [Phenylalanine, tyrosine and tryptophan biosynthesis](file:///C:\Documents%20and%20Settings\Administrator\%25E6%25A1%258C%25E9%259D%25A2\%25E9%2599%2588%25E5%25BB%25BA%25E8%258D%25A3\%25E5%259F%25BA%25E5%259B%25A0%25E7%25BB%2584%25E6%2595%25B0%25E6%258D%25AE\%25E5%259F%25BA%25E5%259B%25A0%25E7%25BB%2584%25E6%2595%25B0%25E6%258D%25AE\HSZP10089\annotation\KEGG\All-Unigene.fa.htm#gene69) | 90 | ko00400 |
| 70 | [Mismatch repair](file:///C:\Documents%20and%20Settings\Administrator\%25E6%25A1%258C%25E9%259D%25A2\%25E9%2599%2588%25E5%25BB%25BA%25E8%258D%25A3\%25E5%259F%25BA%25E5%259B%25A0%25E7%25BB%2584%25E6%2595%25B0%25E6%258D%25AE\%25E5%259F%25BA%25E5%259B%25A0%25E7%25BB%2584%25E6%2595%25B0%25E6%258D%25AE\HSZP10089\annotation\KEGG\All-Unigene.fa.htm#gene70) | 85 | ko03430 |
| 71 | [Diterpenoid biosynthesis](file:///C:\Documents%20and%20Settings\Administrator\%25E6%25A1%258C%25E9%259D%25A2\%25E9%2599%2588%25E5%25BB%25BA%25E8%258D%25A3\%25E5%259F%25BA%25E5%259B%25A0%25E7%25BB%2584%25E6%2595%25B0%25E6%258D%25AE\%25E5%259F%25BA%25E5%259B%25A0%25E7%25BB%2584%25E6%2595%25B0%25E6%258D%25AE\HSZP10089\annotation\KEGG\All-Unigene.fa.htm#gene71) | 81 | ko00904 |
| 72 | [DNA replication](file:///C:\Documents%20and%20Settings\Administrator\%25E6%25A1%258C%25E9%259D%25A2\%25E9%2599%2588%25E5%25BB%25BA%25E8%258D%25A3\%25E5%259F%25BA%25E5%259B%25A0%25E7%25BB%2584%25E6%2595%25B0%25E6%258D%25AE\%25E5%259F%25BA%25E5%259B%25A0%25E7%25BB%2584%25E6%2595%25B0%25E6%258D%25AE\HSZP10089\annotation\KEGG\All-Unigene.fa.htm#gene72) | 79 | ko03030 |
| 73 | [Histidine metabolism](file:///C:\Documents%20and%20Settings\Administrator\%25E6%25A1%258C%25E9%259D%25A2\%25E9%2599%2588%25E5%25BB%25BA%25E8%258D%25A3\%25E5%259F%25BA%25E5%259B%25A0%25E7%25BB%2584%25E6%2595%25B0%25E6%258D%25AE\%25E5%259F%25BA%25E5%259B%25A0%25E7%25BB%2584%25E6%2595%25B0%25E6%258D%25AE\HSZP10089\annotation\KEGG\All-Unigene.fa.htm#gene73) | 76 | ko00340 |
| 74 | [Ubiquinone and other terpenoid-quinone biosynthesis](file:///C:\Documents%20and%20Settings\Administrator\%25E6%25A1%258C%25E9%259D%25A2\%25E9%2599%2588%25E5%25BB%25BA%25E8%258D%25A3\%25E5%259F%25BA%25E5%259B%25A0%25E7%25BB%2584%25E6%2595%25B0%25E6%258D%25AE\%25E5%259F%25BA%25E5%259B%25A0%25E7%25BB%2584%25E6%2595%25B0%25E6%258D%25AE\HSZP10089\annotation\KEGG\All-Unigene.fa.htm#gene74) | 75 | ko00130 |
| 75 | [N-Glycan biosynthesis](file:///C:\Documents%20and%20Settings\Administrator\%25E6%25A1%258C%25E9%259D%25A2\%25E9%2599%2588%25E5%25BB%25BA%25E8%258D%25A3\%25E5%259F%25BA%25E5%259B%25A0%25E7%25BB%2584%25E6%2595%25B0%25E6%258D%25AE\%25E5%259F%25BA%25E5%259B%25A0%25E7%25BB%2584%25E6%2595%25B0%25E6%258D%25AE\HSZP10089\annotation\KEGG\All-Unigene.fa.htm#gene75) | 74 | ko00510 |
| 76 | [Protein export](file:///C:\Documents%20and%20Settings\Administrator\%25E6%25A1%258C%25E9%259D%25A2\%25E9%2599%2588%25E5%25BB%25BA%25E8%258D%25A3\%25E5%259F%25BA%25E5%259B%25A0%25E7%25BB%2584%25E6%2595%25B0%25E6%258D%25AE\%25E5%259F%25BA%25E5%259B%25A0%25E7%25BB%2584%25E6%2595%25B0%25E6%258D%25AE\HSZP10089\annotation\KEGG\All-Unigene.fa.htm#gene76) | 74 | ko03060 |
| 77 | [Nitrogen metabolism](file:///C:\Documents%20and%20Settings\Administrator\%25E6%25A1%258C%25E9%259D%25A2\%25E9%2599%2588%25E5%25BB%25BA%25E8%258D%25A3\%25E5%259F%25BA%25E5%259B%25A0%25E7%25BB%2584%25E6%2595%25B0%25E6%258D%25AE\%25E5%259F%25BA%25E5%259B%25A0%25E7%25BB%2584%25E6%2595%25B0%25E6%258D%25AE\HSZP10089\annotation\KEGG\All-Unigene.fa.htm#gene77) | 74 | ko00910 |
| 78 | [Terpenoid backbone biosynthesis](file:///C:\Documents%20and%20Settings\Administrator\%25E6%25A1%258C%25E9%259D%25A2\%25E9%2599%2588%25E5%25BB%25BA%25E8%258D%25A3\%25E5%259F%25BA%25E5%259B%25A0%25E7%25BB%2584%25E6%2595%25B0%25E6%258D%25AE\%25E5%259F%25BA%25E5%259B%25A0%25E7%25BB%2584%25E6%2595%25B0%25E6%258D%25AE\HSZP10089\annotation\KEGG\All-Unigene.fa.htm#gene78) | 73 | ko00900 |
| 79 | [Natural killer cell mediated cytotoxicity](file:///C:\Documents%20and%20Settings\Administrator\%25E6%25A1%258C%25E9%259D%25A2\%25E9%2599%2588%25E5%25BB%25BA%25E8%258D%25A3\%25E5%259F%25BA%25E5%259B%25A0%25E7%25BB%2584%25E6%2595%25B0%25E6%258D%25AE\%25E5%259F%25BA%25E5%259B%25A0%25E7%25BB%2584%25E6%2595%25B0%25E6%258D%25AE\HSZP10089\annotation\KEGG\All-Unigene.fa.htm#gene79) | 69 | ko04650 |
| 80 | [Proteasome](file:///C:\Documents%20and%20Settings\Administrator\%25E6%25A1%258C%25E9%259D%25A2\%25E9%2599%2588%25E5%25BB%25BA%25E8%258D%25A3\%25E5%259F%25BA%25E5%259B%25A0%25E7%25BB%2584%25E6%2595%25B0%25E6%258D%25AE\%25E5%259F%25BA%25E5%259B%25A0%25E7%25BB%2584%25E6%2595%25B0%25E6%258D%25AE\HSZP10089\annotation\KEGG\All-Unigene.fa.htm#gene80) | 69 | ko03050 |
| 81 | [Flavone and flavonol biosynthesis](file:///C:\Documents%20and%20Settings\Administrator\%25E6%25A1%258C%25E9%259D%25A2\%25E9%2599%2588%25E5%25BB%25BA%25E8%258D%25A3\%25E5%259F%25BA%25E5%259B%25A0%25E7%25BB%2584%25E6%2595%25B0%25E6%258D%25AE\%25E5%259F%25BA%25E5%259B%25A0%25E7%25BB%2584%25E6%2595%25B0%25E6%258D%25AE\HSZP10089\annotation\KEGG\All-Unigene.fa.htm#gene81) | 68 | ko00944 |
| 82 | [SNARE interactions in vesicular transport](file:///C:\Documents%20and%20Settings\Administrator\%25E6%25A1%258C%25E9%259D%25A2\%25E9%2599%2588%25E5%25BB%25BA%25E8%258D%25A3\%25E5%259F%25BA%25E5%259B%25A0%25E7%25BB%2584%25E6%2595%25B0%25E6%258D%25AE\%25E5%259F%25BA%25E5%259B%25A0%25E7%25BB%2584%25E6%2595%25B0%25E6%258D%25AE\HSZP10089\annotation\KEGG\All-Unigene.fa.htm#gene82) | 65 | ko04130 |
| 83 | [Butanoate metabolism](file:///C:\Documents%20and%20Settings\Administrator\%25E6%25A1%258C%25E9%259D%25A2\%25E9%2599%2588%25E5%25BB%25BA%25E8%258D%25A3\%25E5%259F%25BA%25E5%259B%25A0%25E7%25BB%2584%25E6%2595%25B0%25E6%258D%25AE\%25E5%259F%25BA%25E5%259B%25A0%25E7%25BB%2584%25E6%2595%25B0%25E6%258D%25AE\HSZP10089\annotation\KEGG\All-Unigene.fa.htm#gene83) | 63 | ko00650 |
| 84 | [Fatty acid biosynthesis](file:///C:\Documents%20and%20Settings\Administrator\%25E6%25A1%258C%25E9%259D%25A2\%25E9%2599%2588%25E5%25BB%25BA%25E8%258D%25A3\%25E5%259F%25BA%25E5%259B%25A0%25E7%25BB%2584%25E6%2595%25B0%25E6%258D%25AE\%25E5%259F%25BA%25E5%259B%25A0%25E7%25BB%2584%25E6%2595%25B0%25E6%258D%25AE\HSZP10089\annotation\KEGG\All-Unigene.fa.htm#gene84) | 61 | ko00061 |
| 85 | [Other glycan degradation](file:///C:\Documents%20and%20Settings\Administrator\%25E6%25A1%258C%25E9%259D%25A2\%25E9%2599%2588%25E5%25BB%25BA%25E8%258D%25A3\%25E5%259F%25BA%25E5%259B%25A0%25E7%25BB%2584%25E6%2595%25B0%25E6%258D%25AE\%25E5%259F%25BA%25E5%259B%25A0%25E7%25BB%2584%25E6%2595%25B0%25E6%258D%25AE\HSZP10089\annotation\KEGG\All-Unigene.fa.htm#gene85) | 61 | ko00511 |
| 86 | [Glycosylphosphatidylinositol(GPI)-anchor biosynthesis](file:///C:\Documents%20and%20Settings\Administrator\%25E6%25A1%258C%25E9%259D%25A2\%25E9%2599%2588%25E5%25BB%25BA%25E8%258D%25A3\%25E5%259F%25BA%25E5%259B%25A0%25E7%25BB%2584%25E6%2595%25B0%25E6%258D%25AE\%25E5%259F%25BA%25E5%259B%25A0%25E7%25BB%2584%25E6%2595%25B0%25E6%258D%25AE\HSZP10089\annotation\KEGG\All-Unigene.fa.htm#gene86) | 60 | ko00563 |
| 87 | [Valine, leucine and isoleucine biosynthesis](file:///C:\Documents%20and%20Settings\Administrator\%25E6%25A1%258C%25E9%259D%25A2\%25E9%2599%2588%25E5%25BB%25BA%25E8%258D%25A3\%25E5%259F%25BA%25E5%259B%25A0%25E7%25BB%2584%25E6%2595%25B0%25E6%258D%25AE\%25E5%259F%25BA%25E5%259B%25A0%25E7%25BB%2584%25E6%2595%25B0%25E6%258D%25AE\HSZP10089\annotation\KEGG\All-Unigene.fa.htm#gene87) | 59 | ko00290 |
| 88 | [Sphingolipid metabolism](file:///C:\Documents%20and%20Settings\Administrator\%25E6%25A1%258C%25E9%259D%25A2\%25E9%2599%2588%25E5%25BB%25BA%25E8%258D%25A3\%25E5%259F%25BA%25E5%259B%25A0%25E7%25BB%2584%25E6%2595%25B0%25E6%258D%25AE\%25E5%259F%25BA%25E5%259B%25A0%25E7%25BB%2584%25E6%2595%25B0%25E6%258D%25AE\HSZP10089\annotation\KEGG\All-Unigene.fa.htm#gene88) | 56 | ko00600 |
| 89 | [Tropane, piperidine and pyridine alkaloid biosynthesis](file:///C:\Documents%20and%20Settings\Administrator\%25E6%25A1%258C%25E9%259D%25A2\%25E9%2599%2588%25E5%25BB%25BA%25E8%258D%25A3\%25E5%259F%25BA%25E5%259B%25A0%25E7%25BB%2584%25E6%2595%25B0%25E6%258D%25AE\%25E5%259F%25BA%25E5%259B%25A0%25E7%25BB%2584%25E6%2595%25B0%25E6%258D%25AE\HSZP10089\annotation\KEGG\All-Unigene.fa.htm#gene89) | 55 | ko00960 |
| 90 | [Glycosaminoglycan degradation](file:///C:\Documents%20and%20Settings\Administrator\%25E6%25A1%258C%25E9%259D%25A2\%25E9%2599%2588%25E5%25BB%25BA%25E8%258D%25A3\%25E5%259F%25BA%25E5%259B%25A0%25E7%25BB%2584%25E6%2595%25B0%25E6%258D%25AE\%25E5%259F%25BA%25E5%259B%25A0%25E7%25BB%2584%25E6%2595%25B0%25E6%258D%25AE\HSZP10089\annotation\KEGG\All-Unigene.fa.htm#gene90) | 54 | ko00531 |
| 91 | [Benzoxazinoid biosynthesis](file:///C:\Documents%20and%20Settings\Administrator\%25E6%25A1%258C%25E9%259D%25A2\%25E9%2599%2588%25E5%25BB%25BA%25E8%258D%25A3\%25E5%259F%25BA%25E5%259B%25A0%25E7%25BB%2584%25E6%2595%25B0%25E6%258D%25AE\%25E5%259F%25BA%25E5%259B%25A0%25E7%25BB%2584%25E6%2595%25B0%25E6%258D%25AE\HSZP10089\annotation\KEGG\All-Unigene.fa.htm#gene91) | 52 | ko00402 |
| 92 | [Sulfur metabolism](file:///C:\Documents%20and%20Settings\Administrator\%25E6%25A1%258C%25E9%259D%25A2\%25E9%2599%2588%25E5%25BB%25BA%25E8%258D%25A3\%25E5%259F%25BA%25E5%259B%25A0%25E7%25BB%2584%25E6%2595%25B0%25E6%258D%25AE\%25E5%259F%25BA%25E5%259B%25A0%25E7%25BB%2584%25E6%2595%25B0%25E6%258D%25AE\HSZP10089\annotation\KEGG\All-Unigene.fa.htm#gene92) | 52 | ko00920 |
| 93 | [Pantothenate and CoA biosynthesis](file:///C:\Documents%20and%20Settings\Administrator\%25E6%25A1%258C%25E9%259D%25A2\%25E9%2599%2588%25E5%25BB%25BA%25E8%258D%25A3\%25E5%259F%25BA%25E5%259B%25A0%25E7%25BB%2584%25E6%2595%25B0%25E6%258D%25AE\%25E5%259F%25BA%25E5%259B%25A0%25E7%25BB%2584%25E6%2595%25B0%25E6%258D%25AE\HSZP10089\annotation\KEGG\All-Unigene.fa.htm#gene93) | 51 | ko00770 |
| 94 | [Isoquinoline alkaloid biosynthesis](file:///C:\Documents%20and%20Settings\Administrator\%25E6%25A1%258C%25E9%259D%25A2\%25E9%2599%2588%25E5%25BB%25BA%25E8%258D%25A3\%25E5%259F%25BA%25E5%259B%25A0%25E7%25BB%2584%25E6%2595%25B0%25E6%258D%25AE\%25E5%259F%25BA%25E5%259B%25A0%25E7%25BB%2584%25E6%2595%25B0%25E6%258D%25AE\HSZP10089\annotation\KEGG\All-Unigene.fa.htm#gene94) | 43 | ko00950 |
| 95 | [Riboflavin metabolism](file:///C:\Documents%20and%20Settings\Administrator\%25E6%25A1%258C%25E9%259D%25A2\%25E9%2599%2588%25E5%25BB%25BA%25E8%258D%25A3\%25E5%259F%25BA%25E5%259B%25A0%25E7%25BB%2584%25E6%2595%25B0%25E6%258D%25AE\%25E5%259F%25BA%25E5%259B%25A0%25E7%25BB%2584%25E6%2595%25B0%25E6%258D%25AE\HSZP10089\annotation\KEGG\All-Unigene.fa.htm#gene95) | 42 | ko00740 |
| 96 | [Steroid biosynthesis](file:///C:\Documents%20and%20Settings\Administrator\%25E6%25A1%258C%25E9%259D%25A2\%25E9%2599%2588%25E5%25BB%25BA%25E8%258D%25A3\%25E5%259F%25BA%25E5%259B%25A0%25E7%25BB%2584%25E6%2595%25B0%25E6%258D%25AE\%25E5%259F%25BA%25E5%259B%25A0%25E7%25BB%2584%25E6%2595%25B0%25E6%258D%25AE\HSZP10089\annotation\KEGG\All-Unigene.fa.htm#gene96) | 39 | ko00100 |
| 97 | [Linoleic acid metabolism](file:///C:\Documents%20and%20Settings\Administrator\%25E6%25A1%258C%25E9%259D%25A2\%25E9%2599%2588%25E5%25BB%25BA%25E8%258D%25A3\%25E5%259F%25BA%25E5%259B%25A0%25E7%25BB%2584%25E6%2595%25B0%25E6%258D%25AE\%25E5%259F%25BA%25E5%259B%25A0%25E7%25BB%2584%25E6%2595%25B0%25E6%258D%25AE\HSZP10089\annotation\KEGG\All-Unigene.fa.htm#gene97) | 36 | ko00591 |
| 98 | [Selenocompound metabolism](file:///C:\Documents%20and%20Settings\Administrator\%25E6%25A1%258C%25E9%259D%25A2\%25E9%2599%2588%25E5%25BB%25BA%25E8%258D%25A3\%25E5%259F%25BA%25E5%259B%25A0%25E7%25BB%2584%25E6%2595%25B0%25E6%258D%25AE\%25E5%259F%25BA%25E5%259B%25A0%25E7%25BB%2584%25E6%2595%25B0%25E6%258D%25AE\HSZP10089\annotation\KEGG\All-Unigene.fa.htm#gene98) | 35 | ko00450 |
| 99 | [Glycosphingolipid biosynthesis - ganglio series](file:///C:\Documents%20and%20Settings\Administrator\%25E6%25A1%258C%25E9%259D%25A2\%25E9%2599%2588%25E5%25BB%25BA%25E8%258D%25A3\%25E5%259F%25BA%25E5%259B%25A0%25E7%25BB%2584%25E6%2595%25B0%25E6%258D%25AE\%25E5%259F%25BA%25E5%259B%25A0%25E7%25BB%2584%25E6%2595%25B0%25E6%258D%25AE\HSZP10089\annotation\KEGG\All-Unigene.fa.htm#gene99) | 33 | ko00604 |
| 100 | [Lysine biosynthesis](file:///C:\Documents%20and%20Settings\Administrator\%25E6%25A1%258C%25E9%259D%25A2\%25E9%2599%2588%25E5%25BB%25BA%25E8%258D%25A3\%25E5%259F%25BA%25E5%259B%25A0%25E7%25BB%2584%25E6%2595%25B0%25E6%258D%25AE\%25E5%259F%25BA%25E5%259B%25A0%25E7%25BB%2584%25E6%2595%25B0%25E6%258D%25AE\HSZP10089\annotation\KEGG\All-Unigene.fa.htm#gene100) | 33 | ko00300 |
| 101 | [Vitamin B6 metabolism](file:///C:\Documents%20and%20Settings\Administrator\%25E6%25A1%258C%25E9%259D%25A2\%25E9%2599%2588%25E5%25BB%25BA%25E8%258D%25A3\%25E5%259F%25BA%25E5%259B%25A0%25E7%25BB%2584%25E6%2595%25B0%25E6%258D%25AE\%25E5%259F%25BA%25E5%259B%25A0%25E7%25BB%2584%25E6%2595%25B0%25E6%258D%25AE\HSZP10089\annotation\KEGG\All-Unigene.fa.htm#gene101) | 31 | ko00750 |
| 102 | [Folate biosynthesis](file:///C:\Documents%20and%20Settings\Administrator\%25E6%25A1%258C%25E9%259D%25A2\%25E9%2599%2588%25E5%25BB%25BA%25E8%258D%25A3\%25E5%259F%25BA%25E5%259B%25A0%25E7%25BB%2584%25E6%2595%25B0%25E6%258D%25AE\%25E5%259F%25BA%25E5%259B%25A0%25E7%25BB%2584%25E6%2595%25B0%25E6%258D%25AE\HSZP10089\annotation\KEGG\All-Unigene.fa.htm#gene102) | 29 | ko00790 |
| 103 | [One carbon pool by folate](file:///C:\Documents%20and%20Settings\Administrator\%25E6%25A1%258C%25E9%259D%25A2\%25E9%2599%2588%25E5%25BB%25BA%25E8%258D%25A3\%25E5%259F%25BA%25E5%259B%25A0%25E7%25BB%2584%25E6%2595%25B0%25E6%258D%25AE\%25E5%259F%25BA%25E5%259B%25A0%25E7%25BB%2584%25E6%2595%25B0%25E6%258D%25AE\HSZP10089\annotation\KEGG\All-Unigene.fa.htm#gene103) | 29 | ko00670 |
| 104 | [Glucosinolate biosynthesis](file:///C:\Documents%20and%20Settings\Administrator\%25E6%25A1%258C%25E9%259D%25A2\%25E9%2599%2588%25E5%25BB%25BA%25E8%258D%25A3\%25E5%259F%25BA%25E5%259B%25A0%25E7%25BB%2584%25E6%2595%25B0%25E6%258D%25AE\%25E5%259F%25BA%25E5%259B%25A0%25E7%25BB%2584%25E6%2595%25B0%25E6%258D%25AE\HSZP10089\annotation\KEGG\All-Unigene.fa.htm#gene104) | 27 | ko00966 |
| 105 | [Arachidonic acid metabolism](file:///C:\Documents%20and%20Settings\Administrator\%25E6%25A1%258C%25E9%259D%25A2\%25E9%2599%2588%25E5%25BB%25BA%25E8%258D%25A3\%25E5%259F%25BA%25E5%259B%25A0%25E7%25BB%2584%25E6%2595%25B0%25E6%258D%25AE\%25E5%259F%25BA%25E5%259B%25A0%25E7%25BB%2584%25E6%2595%25B0%25E6%258D%25AE\HSZP10089\annotation\KEGG\All-Unigene.fa.htm#gene105) | 25 | ko00590 |
| 106 | [Non-homologous end-joining](file:///C:\Documents%20and%20Settings\Administrator\%25E6%25A1%258C%25E9%259D%25A2\%25E9%2599%2588%25E5%25BB%25BA%25E8%258D%25A3\%25E5%259F%25BA%25E5%259B%25A0%25E7%25BB%2584%25E6%2595%25B0%25E6%258D%25AE\%25E5%259F%25BA%25E5%259B%25A0%25E7%25BB%2584%25E6%2595%25B0%25E6%258D%25AE\HSZP10089\annotation\KEGG\All-Unigene.fa.htm#gene106) | 25 | ko03450 |
| 107 | [Nicotinate and nicotinamide metabolism](file:///C:\Documents%20and%20Settings\Administrator\%25E6%25A1%258C%25E9%259D%25A2\%25E9%2599%2588%25E5%25BB%25BA%25E8%258D%25A3\%25E5%259F%25BA%25E5%259B%25A0%25E7%25BB%2584%25E6%2595%25B0%25E6%258D%25AE\%25E5%259F%25BA%25E5%259B%25A0%25E7%25BB%2584%25E6%2595%25B0%25E6%258D%25AE\HSZP10089\annotation\KEGG\All-Unigene.fa.htm#gene107) | 25 | ko00760 |
| 108 | [Sulfur relay system](file:///C:\Documents%20and%20Settings\Administrator\%25E6%25A1%258C%25E9%259D%25A2\%25E9%2599%2588%25E5%25BB%25BA%25E8%258D%25A3\%25E5%259F%25BA%25E5%259B%25A0%25E7%25BB%2584%25E6%2595%25B0%25E6%258D%25AE\%25E5%259F%25BA%25E5%259B%25A0%25E7%25BB%2584%25E6%2595%25B0%25E6%258D%25AE\HSZP10089\annotation\KEGG\All-Unigene.fa.htm#gene108) | 25 | ko04122 |
| 109 | [Photosynthesis - antenna proteins](file:///C:\Documents%20and%20Settings\Administrator\%25E6%25A1%258C%25E9%259D%25A2\%25E9%2599%2588%25E5%25BB%25BA%25E8%258D%25A3\%25E5%259F%25BA%25E5%259B%25A0%25E7%25BB%2584%25E6%2595%25B0%25E6%258D%25AE\%25E5%259F%25BA%25E5%259B%25A0%25E7%25BB%2584%25E6%2595%25B0%25E6%258D%25AE\HSZP10089\annotation\KEGG\All-Unigene.fa.htm#gene109) | 24 | ko00196 |
| 110 | [Indole alkaloid biosynthesis](file:///C:\Documents%20and%20Settings\Administrator\%25E6%25A1%258C%25E9%259D%25A2\%25E9%2599%2588%25E5%25BB%25BA%25E8%258D%25A3\%25E5%259F%25BA%25E5%259B%25A0%25E7%25BB%2584%25E6%2595%25B0%25E6%258D%25AE\%25E5%259F%25BA%25E5%259B%25A0%25E7%25BB%2584%25E6%2595%25B0%25E6%258D%25AE\HSZP10089\annotation\KEGG\All-Unigene.fa.htm#gene110) | 24 | ko00901 |
| 111 | [Circadian rhythm - mammal](file:///C:\Documents%20and%20Settings\Administrator\%25E6%25A1%258C%25E9%259D%25A2\%25E9%2599%2588%25E5%25BB%25BA%25E8%258D%25A3\%25E5%259F%25BA%25E5%259B%25A0%25E7%25BB%2584%25E6%2595%25B0%25E6%258D%25AE\%25E5%259F%25BA%25E5%259B%25A0%25E7%25BB%2584%25E6%2595%25B0%25E6%258D%25AE\HSZP10089\annotation\KEGG\All-Unigene.fa.htm#gene111) | 22 | ko04710 |
| 112 | [Monoterpenoid biosynthesis](file:///C:\Documents%20and%20Settings\Administrator\%25E6%25A1%258C%25E9%259D%25A2\%25E9%2599%2588%25E5%25BB%25BA%25E8%258D%25A3\%25E5%259F%25BA%25E5%259B%25A0%25E7%25BB%2584%25E6%2595%25B0%25E6%258D%25AE\%25E5%259F%25BA%25E5%259B%25A0%25E7%25BB%2584%25E6%2595%25B0%25E6%258D%25AE\HSZP10089\annotation\KEGG\All-Unigene.fa.htm#gene112) | 20 | ko00902 |
| 113 | [Brassinosteroid biosynthesis](file:///C:\Documents%20and%20Settings\Administrator\%25E6%25A1%258C%25E9%259D%25A2\%25E9%2599%2588%25E5%25BB%25BA%25E8%258D%25A3\%25E5%259F%25BA%25E5%259B%25A0%25E7%25BB%2584%25E6%2595%25B0%25E6%258D%25AE\%25E5%259F%25BA%25E5%259B%25A0%25E7%25BB%2584%25E6%2595%25B0%25E6%258D%25AE\HSZP10089\annotation\KEGG\All-Unigene.fa.htm#gene113) | 20 | ko00905 |
| 114 | [Taurine and hypotaurine metabolism](file:///C:\Documents%20and%20Settings\Administrator\%25E6%25A1%258C%25E9%259D%25A2\%25E9%2599%2588%25E5%25BB%25BA%25E8%258D%25A3\%25E5%259F%25BA%25E5%259B%25A0%25E7%25BB%2584%25E6%2595%25B0%25E6%258D%25AE\%25E5%259F%25BA%25E5%259B%25A0%25E7%25BB%2584%25E6%2595%25B0%25E6%258D%25AE\HSZP10089\annotation\KEGG\All-Unigene.fa.htm#gene114) | 19 | ko00430 |
| 115 | [Other types of O-glycan biosynthesis](file:///C:\Documents%20and%20Settings\Administrator\%25E6%25A1%258C%25E9%259D%25A2\%25E9%2599%2588%25E5%25BB%25BA%25E8%258D%25A3\%25E5%259F%25BA%25E5%259B%25A0%25E7%25BB%2584%25E6%2595%25B0%25E6%258D%25AE\%25E5%259F%25BA%25E5%259B%25A0%25E7%25BB%2584%25E6%2595%25B0%25E6%258D%25AE\HSZP10089\annotation\KEGG\All-Unigene.fa.htm#gene115) | 16 | ko00514 |
| 116 | [Sesquiterpenoid biosynthesis](file:///C:\Documents%20and%20Settings\Administrator\%25E6%25A1%258C%25E9%259D%25A2\%25E9%2599%2588%25E5%25BB%25BA%25E8%258D%25A3\%25E5%259F%25BA%25E5%259B%25A0%25E7%25BB%2584%25E6%2595%25B0%25E6%258D%25AE\%25E5%259F%25BA%25E5%259B%25A0%25E7%25BB%2584%25E6%2595%25B0%25E6%258D%25AE\HSZP10089\annotation\KEGG\All-Unigene.fa.htm#gene116) | 15 | ko00909 |
| 117 | [Thiamine metabolism](file:///C:\Documents%20and%20Settings\Administrator\%25E6%25A1%258C%25E9%259D%25A2\%25E9%2599%2588%25E5%25BB%25BA%25E8%258D%25A3\%25E5%259F%25BA%25E5%259B%25A0%25E7%25BB%2584%25E6%2595%25B0%25E6%258D%25AE\%25E5%259F%25BA%25E5%259B%25A0%25E7%25BB%2584%25E6%2595%25B0%25E6%258D%25AE\HSZP10089\annotation\KEGG\All-Unigene.fa.htm#gene117) | 15 | ko00730 |
| 118 | [Glycosphingolipid biosynthesis - globo series](file:///C:\Documents%20and%20Settings\Administrator\%25E6%25A1%258C%25E9%259D%25A2\%25E9%2599%2588%25E5%25BB%25BA%25E8%258D%25A3\%25E5%259F%25BA%25E5%259B%25A0%25E7%25BB%2584%25E6%2595%25B0%25E6%258D%25AE\%25E5%259F%25BA%25E5%259B%25A0%25E7%25BB%2584%25E6%2595%25B0%25E6%258D%25AE\HSZP10089\annotation\KEGG\All-Unigene.fa.htm#gene118) | 15 | ko00603 |
| 119 | [Synthesis and degradation of ketone bodies](file:///C:\Documents%20and%20Settings\Administrator\%25E6%25A1%258C%25E9%259D%25A2\%25E9%2599%2588%25E5%25BB%25BA%25E8%258D%25A3\%25E5%259F%25BA%25E5%259B%25A0%25E7%25BB%2584%25E6%2595%25B0%25E6%258D%25AE\%25E5%259F%25BA%25E5%259B%25A0%25E7%25BB%2584%25E6%2595%25B0%25E6%258D%25AE\HSZP10089\annotation\KEGG\All-Unigene.fa.htm#gene119) | 9 | ko00072 |
| 120 | [C5-Branched dibasic acid metabolism](file:///C:\Documents%20and%20Settings\Administrator\%25E6%25A1%258C%25E9%259D%25A2\%25E9%2599%2588%25E5%25BB%25BA%25E8%258D%25A3\%25E5%259F%25BA%25E5%259B%25A0%25E7%25BB%2584%25E6%2595%25B0%25E6%258D%25AE\%25E5%259F%25BA%25E5%259B%25A0%25E7%25BB%2584%25E6%2595%25B0%25E6%258D%25AE\HSZP10089\annotation\KEGG\All-Unigene.fa.htm#gene120) | 9 | ko00660 |
| 121 | [Anthocyanin biosynthesis](file:///C:\Documents%20and%20Settings\Administrator\%25E6%25A1%258C%25E9%259D%25A2\%25E9%2599%2588%25E5%25BB%25BA%25E8%258D%25A3\%25E5%259F%25BA%25E5%259B%25A0%25E7%25BB%2584%25E6%2595%25B0%25E6%258D%25AE\%25E5%259F%25BA%25E5%259B%25A0%25E7%25BB%2584%25E6%2595%25B0%25E6%258D%25AE\HSZP10089\annotation\KEGG\All-Unigene.fa.htm#gene121) | 7 | ko00942 |
| 122 | [Fatty acid elongation](file:///C:\Documents%20and%20Settings\Administrator\%25E6%25A1%258C%25E9%259D%25A2\%25E9%2599%2588%25E5%25BB%25BA%25E8%258D%25A3\%25E5%259F%25BA%25E5%259B%25A0%25E7%25BB%2584%25E6%2595%25B0%25E6%258D%25AE\%25E5%259F%25BA%25E5%259B%25A0%25E7%25BB%2584%25E6%2595%25B0%25E6%258D%25AE\HSZP10089\annotation\KEGG\All-Unigene.fa.htm#gene122) | 7 | ko00062 |
| 123 | [Biotin metabolism](file:///C:\Documents%20and%20Settings\Administrator\%25E6%25A1%258C%25E9%259D%25A2\%25E9%2599%2588%25E5%25BB%25BA%25E8%258D%25A3\%25E5%259F%25BA%25E5%259B%25A0%25E7%25BB%2584%25E6%2595%25B0%25E6%258D%25AE\%25E5%259F%25BA%25E5%259B%25A0%25E7%25BB%2584%25E6%2595%25B0%25E6%258D%25AE\HSZP10089\annotation\KEGG\All-Unigene.fa.htm#gene123) | 7 | ko00780 |
| 124 | [Lipoic acid metabolism](file:///C:\Documents%20and%20Settings\Administrator\%25E6%25A1%258C%25E9%259D%25A2\%25E9%2599%2588%25E5%25BB%25BA%25E8%258D%25A3\%25E5%259F%25BA%25E5%259B%25A0%25E7%25BB%2584%25E6%2595%25B0%25E6%258D%25AE\%25E5%259F%25BA%25E5%259B%25A0%25E7%25BB%2584%25E6%2595%25B0%25E6%258D%25AE\HSZP10089\annotation\KEGG\All-Unigene.fa.htm#gene124) | 5 | ko00785 |
| 125 | [Caffeine metabolism](file:///C:\Documents%20and%20Settings\Administrator\%25E6%25A1%258C%25E9%259D%25A2\%25E9%2599%2588%25E5%25BB%25BA%25E8%258D%25A3\%25E5%259F%25BA%25E5%259B%25A0%25E7%25BB%2584%25E6%2595%25B0%25E6%258D%25AE\%25E5%259F%25BA%25E5%259B%25A0%25E7%25BB%2584%25E6%2595%25B0%25E6%258D%25AE\HSZP10089\annotation\KEGG\All-Unigene.fa.htm#gene125) | 3 | ko00232 |
