# Supplementary material for: Transcriptome Sequencing and Profiling of Expressed Genes in Phloem and Xylem of Ramie (Boehmeria nivea L. Gaud)
Source: PLoS One. 2014 Oct 29;9(10):e110623. doi: 10.1371/journal.pone.0110623 (PMC4213010; doi:10.1371/journal.pone.0110623)
Supplement: Table S5 — Pathway assignment of DEGs genes based on KEGG. (DOCX) [file pone.0110623.s005.docx]

Table S4. Pathway assignment of DEGs genes based on KEGG.

| Rank | Pathway | DEGs genes | Pvalue | Pathway ID |
| --- | --- | --- | --- | --- |
| 1 | [Metabolic pathways](file:///C:\Documents%20and%20Settings\Administrator\桌面\新建%20Microsoft%20Office%20Excel%20工作表%20(3).xlsx#RANGE!gene1) | 1950 | 2.6202E-38 | ko01100 |
| 2 | [Ribosome](file:///C:\Documents%20and%20Settings\Administrator\桌面\新建%20Microsoft%20Office%20Excel%20工作表%20(3).xlsx#RANGE!gene2) | 388 | 7.4795E-33 | ko03010 |
| 3 | [Biosynthesis of secondary metabolites](file:///C:\Documents%20and%20Settings\Administrator\桌面\新建%20Microsoft%20Office%20Excel%20工作表%20(3).xlsx#RANGE!gene3) | 918 | 5.7446E-30 | ko01110 |
| 4 | [Phenylpropanoid biosynthesis](file:///C:\Documents%20and%20Settings\Administrator\桌面\新建%20Microsoft%20Office%20Excel%20工作表%20(3).xlsx#RANGE!gene4) | 223 | 1.7103E-28 | ko00940 |
| 5 | [Ether lipid metabolism](file:///C:\Documents%20and%20Settings\Administrator\桌面\新建%20Microsoft%20Office%20Excel%20工作表%20(3).xlsx#RANGE!gene5) | 436 | 5.7513E-17 | ko00565 |
| 6 | [Glycerophospholipid metabolism](file:///C:\Documents%20and%20Settings\Administrator\桌面\新建%20Microsoft%20Office%20Excel%20工作表%20(3).xlsx#RANGE!gene6) | 464 | 4.0104E-15 | ko00564 |
| 7 | [Glycolysis / Gluconeogenesis](file:///C:\Documents%20and%20Settings\Administrator\桌面\新建%20Microsoft%20Office%20Excel%20工作表%20(3).xlsx#RANGE!gene7) | 186 | 2.0127E-14 | ko00010 |
| 8 | [Tryptophan metabolism](file:///C:\Documents%20and%20Settings\Administrator\桌面\新建%20Microsoft%20Office%20Excel%20工作表%20(3).xlsx#RANGE!gene8) | 80 | 2.3107E-14 | ko00380 |
| 9 | [Endocytosis](file:///C:\Documents%20and%20Settings\Administrator\桌面\新建%20Microsoft%20Office%20Excel%20工作表%20(3).xlsx#RANGE!gene9) | 479 | 1.0775E-13 | ko04144 |
| 10 | [Fatty acid metabolism](file:///C:\Documents%20and%20Settings\Administrator\桌面\新建%20Microsoft%20Office%20Excel%20工作表%20(3).xlsx#RANGE!gene10) | 99 | 3.5109E-13 | ko00071 |
| 11 | [Flavonoid biosynthesis](file:///C:\Documents%20and%20Settings\Administrator\桌面\新建%20Microsoft%20Office%20Excel%20工作表%20(3).xlsx#RANGE!gene11) | 127 | 1.3732E-12 | ko00941 |
| 12 | [Stilbenoid, diarylheptanoid and gingerol biosynthesis](file:///C:\Documents%20and%20Settings\Administrator\桌面\新建%20Microsoft%20Office%20Excel%20工作表%20(3).xlsx#RANGE!gene12) | 115 | 5.4364E-12 | ko00945 |
| 13 | [Limonene and pinene degradation](file:///C:\Documents%20and%20Settings\Administrator\桌面\新建%20Microsoft%20Office%20Excel%20工作表%20(3).xlsx#RANGE!gene13) | 114 | 9.3881E-12 | ko00903 |
| 14 | [Glyoxylate and dicarboxylate metabolism](file:///C:\Documents%20and%20Settings\Administrator\桌面\新建%20Microsoft%20Office%20Excel%20工作表%20(3).xlsx#RANGE!gene14) | 77 | 9.4516E-12 | ko00630 |
| 15 | [Plant-pathogen interaction](file:///C:\Documents%20and%20Settings\Administrator\桌面\新建%20Microsoft%20Office%20Excel%20工作表%20(3).xlsx#RANGE!gene15) | 502 | 3.7877E-11 | ko04626 |
| 16 | [Pentose and glucuronate interconversions](file:///C:\Documents%20and%20Settings\Administrator\桌面\新建%20Microsoft%20Office%20Excel%20工作表%20(3).xlsx#RANGE!gene16) | 86 | 3.1358E-10 | ko00040 |
| 17 | [Oxidative phosphorylation](file:///C:\Documents%20and%20Settings\Administrator\桌面\新建%20Microsoft%20Office%20Excel%20工作表%20(3).xlsx#RANGE!gene17) | 176 | 1.0241E-09 | ko00190 |
| 18 | [ABC transporters](file:///C:\Documents%20and%20Settings\Administrator\桌面\新建%20Microsoft%20Office%20Excel%20工作表%20(3).xlsx#RANGE!gene18) | 73 | 4.9695E-09 | ko02010 |
| 19 | [Starch and sucrose metabolism](file:///C:\Documents%20and%20Settings\Administrator\桌面\新建%20Microsoft%20Office%20Excel%20工作表%20(3).xlsx#RANGE!gene19) | 154 | 7.7344E-09 | ko00500 |
| 20 | [RNA polymerase](file:///C:\Documents%20and%20Settings\Administrator\桌面\新建%20Microsoft%20Office%20Excel%20工作表%20(3).xlsx#RANGE!gene20) | 136 | 1.1151E-08 | ko03020 |
| 21 | [Flavone and flavonol biosynthesis](file:///C:\Documents%20and%20Settings\Administrator\桌面\新建%20Microsoft%20Office%20Excel%20工作表%20(3).xlsx#RANGE!gene21) | 44 | 8.9565E-08 | ko00944 |
| 22 | [Ascorbate and aldarate metabolism](file:///C:\Documents%20and%20Settings\Administrator\桌面\新建%20Microsoft%20Office%20Excel%20工作表%20(3).xlsx#RANGE!gene22) | 77 | 1.5497E-07 | ko00053 |
| 23 | [Pentose phosphate pathway](file:///C:\Documents%20and%20Settings\Administrator\桌面\新建%20Microsoft%20Office%20Excel%20工作表%20(3).xlsx#RANGE!gene23) | 95 | 6.8155E-07 | ko00030 |
| 24 | [Phenylalanine metabolism](file:///C:\Documents%20and%20Settings\Administrator\桌面\新建%20Microsoft%20Office%20Excel%20工作表%20(3).xlsx#RANGE!gene24) | 81 | 1.6152E-06 | ko00360 |
| 25 | [Carotenoid biosynthesis](file:///C:\Documents%20and%20Settings\Administrator\桌面\新建%20Microsoft%20Office%20Excel%20工作表%20(3).xlsx#RANGE!gene25) | 59 | 3.0659E-06 | ko00906 |
| 26 | [Diterpenoid biosynthesis](file:///C:\Documents%20and%20Settings\Administrator\桌面\新建%20Microsoft%20Office%20Excel%20工作表%20(3).xlsx#RANGE!gene26) | 47 | 3.3453E-06 | ko00904 |
| 27 | [Tyrosine metabolism](file:///C:\Documents%20and%20Settings\Administrator\桌面\新建%20Microsoft%20Office%20Excel%20工作表%20(3).xlsx#RANGE!gene27) | 68 | 4.3828E-06 | ko00350 |
| 28 | [Cyanoamino acid metabolism](file:///C:\Documents%20and%20Settings\Administrator\桌面\新建%20Microsoft%20Office%20Excel%20工作表%20(3).xlsx#RANGE!gene28) | 65 | 4.5088E-06 | ko00460 |
| 29 | [Glycerolipid metabolism](file:///C:\Documents%20and%20Settings\Administrator\桌面\新建%20Microsoft%20Office%20Excel%20工作表%20(3).xlsx#RANGE!gene29) | 73 | 6.291E-06 | ko00561 |
| 30 | [Plant hormone signal transduction](file:///C:\Documents%20and%20Settings\Administrator\桌面\新建%20Microsoft%20Office%20Excel%20工作表%20(3).xlsx#RANGE!gene30) | 384 | 9.8506E-06 | ko04075 |
| 31 | [Pyruvate metabolism](file:///C:\Documents%20and%20Settings\Administrator\桌面\新建%20Microsoft%20Office%20Excel%20工作表%20(3).xlsx#RANGE!gene31) | 103 | 4.5242E-05 | ko00620 |
| 32 | [Nitrogen metabolism](file:///C:\Documents%20and%20Settings\Administrator\桌面\新建%20Microsoft%20Office%20Excel%20工作表%20(3).xlsx#RANGE!gene32) | 41 | 6.1558E-05 | ko00910 |
| 33 | [Glutathione metabolism](file:///C:\Documents%20and%20Settings\Administrator\桌面\新建%20Microsoft%20Office%20Excel%20工作表%20(3).xlsx#RANGE!gene33) | 77 | 8.8172E-05 | ko00480 |
| 34 | [Citrate cycle (TCA cycle)](file:///C:\Documents%20and%20Settings\Administrator\桌面\新建%20Microsoft%20Office%20Excel%20工作表%20(3).xlsx#RANGE!gene34) | 63 | 0.00010711 | ko00020 |
| 35 | [Benzoxazinoid biosynthesis](file:///C:\Documents%20and%20Settings\Administrator\桌面\新建%20Microsoft%20Office%20Excel%20工作表%20(3).xlsx#RANGE!gene35) | 29 | 0.00061599 | ko00402 |
| 36 | [Arginine and proline metabolism](file:///C:\Documents%20and%20Settings\Administrator\桌面\新建%20Microsoft%20Office%20Excel%20工作表%20(3).xlsx#RANGE!gene36) | 77 | 0.00087491 | ko00330 |
| 37 | [Carbon fixation in photosynthetic organisms](file:///C:\Documents%20and%20Settings\Administrator\桌面\新建%20Microsoft%20Office%20Excel%20工作表%20(3).xlsx#RANGE!gene37) | 74 | 0.00125696 | ko00710 |
| 38 | [Purine metabolism](file:///C:\Documents%20and%20Settings\Administrator\桌面\新建%20Microsoft%20Office%20Excel%20工作表%20(3).xlsx#RANGE!gene38) | 195 | 0.00128066 | ko00230 |
| 39 | [Glucosinolate biosynthesis](file:///C:\Documents%20and%20Settings\Administrator\桌面\新建%20Microsoft%20Office%20Excel%20工作表%20(3).xlsx#RANGE!gene39) | 17 | 0.00138331 | ko00966 |
| 40 | [Valine, leucine and isoleucine degradation](file:///C:\Documents%20and%20Settings\Administrator\桌面\新建%20Microsoft%20Office%20Excel%20工作表%20(3).xlsx#RANGE!gene40) | 57 | 0.00146147 | ko00280 |
| 41 | [Propanoate metabolism](file:///C:\Documents%20and%20Settings\Administrator\桌面\新建%20Microsoft%20Office%20Excel%20工作表%20(3).xlsx#RANGE!gene41) | 58 | 0.00203852 | ko00640 |
| 42 | [Monoterpenoid biosynthesis](file:///C:\Documents%20and%20Settings\Administrator\桌面\新建%20Microsoft%20Office%20Excel%20工作表%20(3).xlsx#RANGE!gene42) | 13 | 0.00342544 | ko00902 |
| 43 | [beta-Alanine metabolism](file:///C:\Documents%20and%20Settings\Administrator\桌面\新建%20Microsoft%20Office%20Excel%20工作表%20(3).xlsx#RANGE!gene43) | 53 | 0.00382151 | ko00410 |
| 44 | [Biosynthesis of unsaturated fatty acids](file:///C:\Documents%20and%20Settings\Administrator\桌面\新建%20Microsoft%20Office%20Excel%20工作表%20(3).xlsx#RANGE!gene44) | 49 | 0.00405881 | ko01040 |
| 45 | [Galactose metabolism](file:///C:\Documents%20and%20Settings\Administrator\桌面\新建%20Microsoft%20Office%20Excel%20工作表%20(3).xlsx#RANGE!gene45) | 73 | 0.00520248 | ko00052 |
| 46 | [alpha-Linolenic acid metabolism](file:///C:\Documents%20and%20Settings\Administrator\桌面\新建%20Microsoft%20Office%20Excel%20工作表%20(3).xlsx#RANGE!gene46) | 53 | 0.00583633 | ko00592 |
| 47 | [Histidine metabolism](file:///C:\Documents%20and%20Settings\Administrator\桌面\新建%20Microsoft%20Office%20Excel%20工作表%20(3).xlsx#RANGE!gene47) | 36 | 0.0066125 | ko00340 |
| 48 | [Peroxisome](file:///C:\Documents%20and%20Settings\Administrator\桌面\新建%20Microsoft%20Office%20Excel%20工作表%20(3).xlsx#RANGE!gene48) | 91 | 0.00675526 | ko04146 |
| 49 | [Alanine, aspartate and glutamate metabolism](file:///C:\Documents%20and%20Settings\Administrator\桌面\新建%20Microsoft%20Office%20Excel%20工作表%20(3).xlsx#RANGE!gene49) | 50 | 0.00843427 | ko00250 |
| 50 | [Zeatin biosynthesis](file:///C:\Documents%20and%20Settings\Administrator\桌面\新建%20Microsoft%20Office%20Excel%20工作表%20(3).xlsx#RANGE!gene50) | 69 | 0.01121962 | ko00908 |
| 51 | [Butanoate metabolism](file:///C:\Documents%20and%20Settings\Administrator\桌面\新建%20Microsoft%20Office%20Excel%20工作表%20(3).xlsx#RANGE!gene51) | 30 | 0.01143715 | ko00650 |
| 52 | [Fructose and mannose metabolism](file:///C:\Documents%20and%20Settings\Administrator\桌面\新建%20Microsoft%20Office%20Excel%20工作表%20(3).xlsx#RANGE!gene52) | 70 | 0.0137113 | ko00051 |
| 53 | [Lysine degradation](file:///C:\Documents%20and%20Settings\Administrator\桌面\新建%20Microsoft%20Office%20Excel%20工作表%20(3).xlsx#RANGE!gene53) | 42 | 0.01510476 | ko00310 |
| 54 | [Pyrimidine metabolism](file:///C:\Documents%20and%20Settings\Administrator\桌面\新建%20Microsoft%20Office%20Excel%20工作表%20(3).xlsx#RANGE!gene54) | 161 | 0.0194735 | ko00240 |
| 55 | [Indole alkaloid biosynthesis](file:///C:\Documents%20and%20Settings\Administrator\桌面\新建%20Microsoft%20Office%20Excel%20工作表%20(3).xlsx#RANGE!gene55) | 13 | 0.02654684 | ko00901 |
| 56 | [Photosynthesis](file:///C:\Documents%20and%20Settings\Administrator\桌面\新建%20Microsoft%20Office%20Excel%20工作表%20(3).xlsx#RANGE!gene56) | 39 | 0.0453448 | ko00195 |
| 57 | [Sesquiterpenoid biosynthesis](file:///C:\Documents%20and%20Settings\Administrator\桌面\新建%20Microsoft%20Office%20Excel%20工作表%20(3).xlsx#RANGE!gene57) | 8 | 0.08456256 | ko00909 |
| 58 | [Brassinosteroid biosynthesis](file:///C:\Documents%20and%20Settings\Administrator\桌面\新建%20Microsoft%20Office%20Excel%20工作表%20(3).xlsx#RANGE!gene58) | 10 | 0.08755464 | ko00905 |
| 59 | [Vitamin B6 metabolism](file:///C:\Documents%20and%20Settings\Administrator\桌面\新建%20Microsoft%20Office%20Excel%20工作表%20(3).xlsx#RANGE!gene59) | 14 | 0.1088717 | ko00750 |
| 60 | [Tropane, piperidine and pyridine alkaloid biosynthesis](file:///C:\Documents%20and%20Settings\Administrator\桌面\新建%20Microsoft%20Office%20Excel%20工作表%20(3).xlsx#RANGE!gene60) | 23 | 0.1091991 | ko00960 |
| 61 | [Fatty acid biosynthesis](file:///C:\Documents%20and%20Settings\Administrator\桌面\新建%20Microsoft%20Office%20Excel%20工作表%20(3).xlsx#RANGE!gene61) | 25 | 0.1200129 | ko00061 |
| 62 | [Cysteine and methionine metabolism](file:///C:\Documents%20and%20Settings\Administrator\桌面\新建%20Microsoft%20Office%20Excel%20工作表%20(3).xlsx#RANGE!gene62) | 63 | 0.1268526 | ko00270 |
| 63 | [Taurine and hypotaurine metabolism](file:///C:\Documents%20and%20Settings\Administrator\桌面\新建%20Microsoft%20Office%20Excel%20工作表%20(3).xlsx#RANGE!gene63) | 9 | 0.1403098 | ko00430 |
| 64 | [Linoleic acid metabolism](file:///C:\Documents%20and%20Settings\Administrator\桌面\新建%20Microsoft%20Office%20Excel%20工作表%20(3).xlsx#RANGE!gene64) | 15 | 0.1779025 | ko00591 |
| 65 | [Lysine biosynthesis](file:///C:\Documents%20and%20Settings\Administrator\桌面\新建%20Microsoft%20Office%20Excel%20工作表%20(3).xlsx#RANGE!gene65) | 13 | 0.2740489 | ko00300 |
| 66 | [Isoquinoline alkaloid biosynthesis](file:///C:\Documents%20and%20Settings\Administrator\桌面\新建%20Microsoft%20Office%20Excel%20工作表%20(3).xlsx#RANGE!gene66) | 16 | 0.3336213 | ko00950 |
| 67 | [Terpenoid backbone biosynthesis](file:///C:\Documents%20and%20Settings\Administrator\桌面\新建%20Microsoft%20Office%20Excel%20工作表%20(3).xlsx#RANGE!gene67) | 25 | 0.4588441 | ko00900 |
| 68 | [Selenocompound metabolism](file:///C:\Documents%20and%20Settings\Administrator\桌面\新建%20Microsoft%20Office%20Excel%20工作表%20(3).xlsx#RANGE!gene68) | 12 | 0.5024648 | ko00450 |
| 69 | [Amino sugar and nucleotide sugar metabolism](file:///C:\Documents%20and%20Settings\Administrator\桌面\新建%20Microsoft%20Office%20Excel%20工作表%20(3).xlsx#RANGE!gene69) | 77 | 0.5093252 | ko00520 |
| 70 | [Steroid biosynthesis](file:///C:\Documents%20and%20Settings\Administrator\桌面\新建%20Microsoft%20Office%20Excel%20工作表%20(3).xlsx#RANGE!gene70) | 13 | 0.5461469 | ko00100 |
| 71 | [Glycosphingolipid biosynthesis - ganglio series](file:///C:\Documents%20and%20Settings\Administrator\桌面\新建%20Microsoft%20Office%20Excel%20工作表%20(3).xlsx#RANGE!gene71) | 11 | 0.5524386 | ko00604 |
| 72 | [Glycosaminoglycan degradation](file:///C:\Documents%20and%20Settings\Administrator\桌面\新建%20Microsoft%20Office%20Excel%20工作表%20(3).xlsx#RANGE!gene72) | 17 | 0.6475712 | ko00531 |
| 73 | [Caffeine metabolism](file:///C:\Documents%20and%20Settings\Administrator\桌面\新建%20Microsoft%20Office%20Excel%20工作表%20(3).xlsx#RANGE!gene73) | 1 | 0.7001984 | ko00232 |
| 74 | [Ubiquinone and other terpenoid-quinone biosynthesis](file:///C:\Documents%20and%20Settings\Administrator\桌面\新建%20Microsoft%20Office%20Excel%20工作表%20(3).xlsx#RANGE!gene74) | 23 | 0.7109974 | ko00130 |
| 75 | [Anthocyanin biosynthesis](file:///C:\Documents%20and%20Settings\Administrator\桌面\新建%20Microsoft%20Office%20Excel%20工作表%20(3).xlsx#RANGE!gene75) | 2 | 0.7317857 | ko00942 |
| 76 | [Fatty acid elongation](file:///C:\Documents%20and%20Settings\Administrator\桌面\新建%20Microsoft%20Office%20Excel%20工作表%20(3).xlsx#RANGE!gene76) | 2 | 0.7317857 | ko00062 |
| 77 | [Glycine, serine and threonine metabolism](file:///C:\Documents%20and%20Settings\Administrator\桌面\新建%20Microsoft%20Office%20Excel%20工作表%20(3).xlsx#RANGE!gene77) | 29 | 0.7356369 | ko00260 |
| 78 | [Inositol phosphate metabolism](file:///C:\Documents%20and%20Settings\Administrator\桌面\新建%20Microsoft%20Office%20Excel%20工作表%20(3).xlsx#RANGE!gene78) | 38 | 0.7471136 | ko00562 |
| 79 | [Sulfur relay system](file:///C:\Documents%20and%20Settings\Administrator\桌面\新建%20Microsoft%20Office%20Excel%20工作表%20(3).xlsx#RANGE!gene79) | 7 | 0.7702972 | ko04122 |
| 80 | [Sphingolipid metabolism](file:///C:\Documents%20and%20Settings\Administrator\桌面\新建%20Microsoft%20Office%20Excel%20工作表%20(3).xlsx#RANGE!gene80) | 16 | 0.803539 | ko00600 |
| 81 | [Synthesis and degradation of ketone bodies](file:///C:\Documents%20and%20Settings\Administrator\桌面\新建%20Microsoft%20Office%20Excel%20工作表%20(3).xlsx#RANGE!gene81) | 2 | 0.8532563 | ko00072 |
| 82 | [C5-Branched dibasic acid metabolism](file:///C:\Documents%20and%20Settings\Administrator\桌面\新建%20Microsoft%20Office%20Excel%20工作表%20(3).xlsx#RANGE!gene82) | 2 | 0.8532563 | ko00660 |
| 83 | [Nicotinate and nicotinamide metabolism](file:///C:\Documents%20and%20Settings\Administrator\桌面\新建%20Microsoft%20Office%20Excel%20工作表%20(3).xlsx#RANGE!gene83) | 6 | 0.8829168 | ko00760 |
| 84 | [Circadian rhythm - mammal](file:///C:\Documents%20and%20Settings\Administrator\桌面\新建%20Microsoft%20Office%20Excel%20工作表%20(3).xlsx#RANGE!gene84) | 5 | 0.8995512 | ko04710 |
| 85 | [Pantothenate and CoA biosynthesis](file:///C:\Documents%20and%20Settings\Administrator\桌面\新建%20Microsoft%20Office%20Excel%20工作表%20(3).xlsx#RANGE!gene85) | 13 | 0.9058505 | ko00770 |
| 86 | [Phosphatidylinositol signaling system](file:///C:\Documents%20and%20Settings\Administrator\桌面\新建%20Microsoft%20Office%20Excel%20工作表%20(3).xlsx#RANGE!gene86) | 33 | 0.9114398 | ko04070 |
| 87 | [Natural killer cell mediated cytotoxicity](file:///C:\Documents%20and%20Settings\Administrator\桌面\新建%20Microsoft%20Office%20Excel%20工作表%20(3).xlsx#RANGE!gene87) | 18 | 0.9159584 | ko04650 |
| 88 | [Thiamine metabolism](file:///C:\Documents%20and%20Settings\Administrator\桌面\新建%20Microsoft%20Office%20Excel%20工作表%20(3).xlsx#RANGE!gene88) | 3 | 0.9175981 | ko00730 |
| 89 | [Glycosphingolipid biosynthesis - globo series](file:///C:\Documents%20and%20Settings\Administrator\桌面\新建%20Microsoft%20Office%20Excel%20工作表%20(3).xlsx#RANGE!gene89) | 3 | 0.9175981 | ko00603 |
| 90 | [Valine, leucine and isoleucine biosynthesis](file:///C:\Documents%20and%20Settings\Administrator\桌面\新建%20Microsoft%20Office%20Excel%20工作表%20(3).xlsx#RANGE!gene90) | 15 | 0.9202576 | ko00290 |
| 91 | [Arachidonic acid metabolism](file:///C:\Documents%20and%20Settings\Administrator\桌面\新建%20Microsoft%20Office%20Excel%20工作表%20(3).xlsx#RANGE!gene91) | 5 | 0.9512472 | ko00590 |
| 92 | [mRNA surveillance pathway](file:///C:\Documents%20and%20Settings\Administrator\桌面\新建%20Microsoft%20Office%20Excel%20工作表%20(3).xlsx#RANGE!gene92) | 262 | 0.9643313 | ko03015 |
| 93 | [Riboflavin metabolism](file:///C:\Documents%20and%20Settings\Administrator\桌面\新建%20Microsoft%20Office%20Excel%20工作表%20(3).xlsx#RANGE!gene93) | 9 | 0.9658608 | ko00740 |
| 94 | [Protein processing in endoplasmic reticulum](file:///C:\Documents%20and%20Settings\Administrator\桌面\新建%20Microsoft%20Office%20Excel%20工作表%20(3).xlsx#RANGE!gene94) | 156 | 0.9667863 | ko04141 |
| 95 | [Phenylalanine, tyrosine and tryptophan biosynthesis](file:///C:\Documents%20and%20Settings\Administrator\桌面\新建%20Microsoft%20Office%20Excel%20工作表%20(3).xlsx#RANGE!gene95) | 22 | 0.9708818 | ko00400 |
| 96 | [RNA transport](file:///C:\Documents%20and%20Settings\Administrator\桌面\新建%20Microsoft%20Office%20Excel%20工作表%20(3).xlsx#RANGE!gene96) | 384 | 0.9766449 | ko03013 |
| 97 | [Photosynthesis - antenna proteins](file:///C:\Documents%20and%20Settings\Administrator\桌面\新建%20Microsoft%20Office%20Excel%20工作表%20(3).xlsx#RANGE!gene97) | 4 | 0.9788794 | ko00196 |
| 98 | [Non-homologous end-joining](file:///C:\Documents%20and%20Settings\Administrator\桌面\新建%20Microsoft%20Office%20Excel%20工作表%20(3).xlsx#RANGE!gene98) | 4 | 0.9841427 | ko03450 |
| 99 | [Other glycan degradation](file:///C:\Documents%20and%20Settings\Administrator\桌面\新建%20Microsoft%20Office%20Excel%20工作表%20(3).xlsx#RANGE!gene99) | 13 | 0.9846518 | ko00511 |
| 100 | [SNARE interactions in vesicular transport](file:///C:\Documents%20and%20Settings\Administrator\桌面\新建%20Microsoft%20Office%20Excel%20工作表%20(3).xlsx#RANGE!gene100) | 14 | 0.9853738 | ko04130 |
| 101 | [Other types of O-glycan biosynthesis](file:///C:\Documents%20and%20Settings\Administrator\桌面\新建%20Microsoft%20Office%20Excel%20工作表%20(3).xlsx#RANGE!gene101) | 2 | 0.9855891 | ko00514 |
| 102 | [Porphyrin and chlorophyll metabolism](file:///C:\Documents%20and%20Settings\Administrator\桌面\新建%20Microsoft%20Office%20Excel%20工作表%20(3).xlsx#RANGE!gene102) | 23 | 0.9912932 | ko00860 |
| 103 | [Base excision repair](file:///C:\Documents%20and%20Settings\Administrator\桌面\新建%20Microsoft%20Office%20Excel%20工作表%20(3).xlsx#RANGE!gene103) | 21 | 0.9930853 | ko03410 |
| 104 | [Phagosome](file:///C:\Documents%20and%20Settings\Administrator\桌面\新建%20Microsoft%20Office%20Excel%20工作表%20(3).xlsx#RANGE!gene104) | 56 | 0.9970675 | ko04145 |
| 105 | [Circadian rhythm - plant](file:///C:\Documents%20and%20Settings\Administrator\桌面\新建%20Microsoft%20Office%20Excel%20工作表%20(3).xlsx#RANGE!gene105) | 41 | 0.9976957 | ko04712 |
| 106 | [Regulation of autophagy](file:///C:\Documents%20and%20Settings\Administrator\桌面\新建%20Microsoft%20Office%20Excel%20工作表%20(3).xlsx#RANGE!gene106) | 22 | 0.998526 | ko04140 |
| 107 | [Folate biosynthesis](file:///C:\Documents%20and%20Settings\Administrator\桌面\新建%20Microsoft%20Office%20Excel%20工作表%20(3).xlsx#RANGE!gene107) | 3 | 0.9990031 | ko00790 |
| 108 | [One carbon pool by folate](file:///C:\Documents%20and%20Settings\Administrator\桌面\新建%20Microsoft%20Office%20Excel%20工作表%20(3).xlsx#RANGE!gene108) | 3 | 0.9990031 | ko00670 |
| 109 | [Homologous recombination](file:///C:\Documents%20and%20Settings\Administrator\桌面\新建%20Microsoft%20Office%20Excel%20工作表%20(3).xlsx#RANGE!gene109) | 19 | 0.9996985 | ko03440 |
| 110 | [Ribosome biogenesis in eukaryotes](file:///C:\Documents%20and%20Settings\Administrator\桌面\新建%20Microsoft%20Office%20Excel%20工作表%20(3).xlsx#RANGE!gene110) | 81 | 0.9999412 | ko03008 |
| 111 | [Ubiquitin mediated proteolysis](file:///C:\Documents%20and%20Settings\Administrator\桌面\新建%20Microsoft%20Office%20Excel%20工作表%20(3).xlsx#RANGE!gene111) | 70 | 0.9999574 | ko04120 |
| 112 | [N-Glycan biosynthesis](file:///C:\Documents%20and%20Settings\Administrator\桌面\新建%20Microsoft%20Office%20Excel%20工作表%20(3).xlsx#RANGE!gene112) | 10 | 0.999968 | ko00510 |
| 113 | [Protein export](file:///C:\Documents%20and%20Settings\Administrator\桌面\新建%20Microsoft%20Office%20Excel%20工作表%20(3).xlsx#RANGE!gene113) | 10 | 0.999968 | ko03060 |
| 114 | [Glycosylphosphatidylinositol(GPI)-anchor biosynthesis](file:///C:\Documents%20and%20Settings\Administrator\桌面\新建%20Microsoft%20Office%20Excel%20工作表%20(3).xlsx#RANGE!gene114) | 7 | 0.999969 | ko00563 |
| 115 | [DNA replication](file:///C:\Documents%20and%20Settings\Administrator\桌面\新建%20Microsoft%20Office%20Excel%20工作表%20(3).xlsx#RANGE!gene115) | 11 | 0.999972 | ko03030 |
| 116 | [Basal transcription factors](file:///C:\Documents%20and%20Settings\Administrator\桌面\新建%20Microsoft%20Office%20Excel%20工作表%20(3).xlsx#RANGE!gene116) | 26 | 0.999973 | ko03022 |
| 117 | [Spliceosome](file:///C:\Documents%20and%20Settings\Administrator\桌面\新建%20Microsoft%20Office%20Excel%20工作表%20(3).xlsx#RANGE!gene117) | 212 | 0.9999901 | ko03040 |
| 118 | [Proteasome](file:///C:\Documents%20and%20Settings\Administrator\桌面\新建%20Microsoft%20Office%20Excel%20工作表%20(3).xlsx#RANGE!gene118) | 8 | 0.999991 | ko03050 |
| 119 | [Sulfur metabolism](file:///C:\Documents%20and%20Settings\Administrator\桌面\新建%20Microsoft%20Office%20Excel%20工作表%20(3).xlsx#RANGE!gene119) | 4 | 0.9999975 | ko00920 |
| 120 | [Mismatch repair](file:///C:\Documents%20and%20Settings\Administrator\桌面\新建%20Microsoft%20Office%20Excel%20工作表%20(3).xlsx#RANGE!gene120) | 10 | 0.9999987 | ko03430 |
| 121 | [Aminoacyl-tRNA biosynthesis](file:///C:\Documents%20and%20Settings\Administrator\桌面\新建%20Microsoft%20Office%20Excel%20工作表%20(3).xlsx#RANGE!gene121) | 14 | 0.9999993 | ko00970 |
| 122 | [Nucleotide excision repair](file:///C:\Documents%20and%20Settings\Administrator\桌面\新建%20Microsoft%20Office%20Excel%20工作表%20(3).xlsx#RANGE!gene122) | 22 | 0.9999997 | ko03420 |
| 123 | [RNA degradation](file:///C:\Documents%20and%20Settings\Administrator\桌面\新建%20Microsoft%20Office%20Excel%20工作表%20(3).xlsx#RANGE!gene123) | 55 | 1 | ko03018 |
